# Physiological stressors and invasive plant infections alter the small RNA transcriptome of the rice blast fungus, *Magnaporthe oryzae*

**DOI:** 10.1186/1471-2164-14-326

**Published:** 2013-05-12

**Authors:** Vidhyavathi Raman, Stacey A Simon, Amanda Romag, Feray Demirci, Sandra M Mathioni, Jixian Zhai, Blake C Meyers, Nicole M Donofrio

**Affiliations:** 1 Department of Plant & Soil Sciences, University of Delaware, Newark, DE, 19716, USA; 2 Delaware Biotechnology Institute, University of Delaware, Newark, DE, 19711, USA; 3 Boyce Thompson Institute, Ithaca, NY, 14853, USA; 4 Department of Phytopathology, Federal University of Lavras, Lavras, MG, 37.200-000, Brazil

**Keywords:** Small RNA, *Magnaporthe oryzae*, Rice blast fungus, siRNA

## Abstract

**Background:**

The rice blast fungus, *Magnaporthe oryzae* is a destructive pathogen of rice and other related crops, causing significant yield losses worldwide. Endogenous small RNAs (sRNAs), including small interfering RNAs (siRNAs) and microRNAs (miRNAs) are critical components of gene regulation in many eukaryotic organisms. Recently several new species of sRNAs have been identified in fungi. This fact along with the availability of genome sequence makes *M. oryzae* a compelling target for sRNA profiling. We have examined sRNA species and their biosynthetic genes in *M. oryzae*, and the degree to which these elements regulate fungal stress responses. To this end, we have characterized sRNAs under different physiological stress conditions, which had not yet been examined in this fungus.

**Results:**

The resulting libraries are composed of more than 37 million total genome matched reads mapping to intergenic regions, coding sequences, retrotransposons, inverted, tandem, and other repeated regions of the genome with more than half of the small RNAs arising from intergenic regions. The 24 nucleotide (nt) size class of sRNAs was predominant. A comparison to transcriptional data of *M. oryzae* undergoing the same physiological stresses indicates that sRNAs play a role in transcriptional regulation for a small subset of genes. Support for this idea comes from generation and characterization of mutants putatively involved in sRNAs biogenesis; our results indicate that the deletion of Dicer-like genes and an RNA-Dependent RNA Polymerase gene increases the transcriptional regulation of this subset of genes, including one involved in virulence.

**Conclusions:**

Various physiological stressors and *in planta* conditions alter the small RNA profile of the rice blast fungus. Characterization of sRNA biosynthetic mutants helps to clarify the role of sRNAs in transcriptional control.

## Background

RNA silencing, the RNA-mediated suppression of a target gene in a sequence specific manner, has been described in many organisms as post-transcriptional gene silencing, RNA interference, and quelling, in plants, animals, and fungi, respectively [1,2]. These silencing pathways are involved in transposon silencing, viral defense, DNA elimination, heterochromatin formation, and post-transcriptional repression of cellular genes [3,4]. The critical steps of these pathways include production of small RNAs (sRNAs) of 19–27 nucleotide (nt) from structured or double-stranded RNA by RNase III-like endonucleases called Dicers, followed by loading of these sRNAs into Argonaute-containing complexes to form RNA-induced silencing complexes (RISC) that guide the cleavage of target transcripts [3,5].

*Magnaporthe oryzae* (hereafter *M. oryzae*), a filamentous fungal pathogen that causes rice blast disease, leads to severe yield losses in cultivated rice worldwide. The *Magnaporthe*-rice pathosystem is an excellent model to study the molecular basis of pathogenesis, in part because of the economic importance and molecular and genetic tractability of both host and pathogen, availability of genome sequences and transcriptome data, and the similarity of the rice blast fungus with other cereal diseases in terms of appressorium formation and intra-cellular tissue invasion [6]–[8]. The blast fungus infects rice plants at all stages and all tissues – leaves, stems, nodes, panicles and roots [8,9]. Leaf infection starts with reversible attachment of conidia to the host surface. The release of spore tip mucilage from conidia forms an irreversible attachment to the surface [7]. Conidia then germinate and form infection structures called appressoria at the end of the germ tubes. Appressoria generate enormous amounts of turgor pressure to penetrate the outer surface of the plant, which is followed by tissue invasion and colonization resulting in disease lesions [8].

The carefully controlled and timed life cycle of *M. oryzae* warrants examination into both transcriptional and post-transcriptional gene regulation. To date, endogenous small RNA species have been examined in several fungal species including the budding yeast, *Saccharomyces cerevisiae*, the model ascomycete fungus and close relative of *M. oryzae*, *Neurospora crassa* and *M. oryzae* itself ([5]; reviewed in [10]–[12]). The *M. oryzae* genome encodes putative RNAi pathway components consisting of two Dicer-like proteins (DCL), three Argonautes (AGO), three RNA-dependent RNA polymerases (RdRP) and one exportin-5 [11,13,14]. In 2004, Kadotani et al. showed that Magnaporthe Dicer-like-2 (MoDcl2) is necessary for generating siRNAs in hairpin dsRNA-mediated RNA silencing, unlike their counterparts in *Neurospora crassa* in which both of the Dicers are redundantly involved in the silencing process [15]. While the Kadotani et al. study (2004) did not find a clear role for the Magnaporthe Dicer-like-1 (MoDcl1) in the generation of siRNAs, the function of this gene is yet to be discovered. Exportin-5, which plays a role in nuclear export of pre-miRNAs in animals, was identified and deleted in *M. oryzae* and shown to have reduced pathogenicity on roots, and to complement its yeast homolog, the nucleocytoplasmic transporter Msn5 [16]–[18].

The complexity of RNAi components in this fungus suggests the possible existence of multiple sRNA pathways. Though miRNAs have not been identified in *M. oryzae*, the published data have demonstrated numerous components of the *M. oryzae* sRNA repertoire, including small interfering (siRNA) -induced silencing of transgenes [11], the presence of viral derived siRNAs [19], methylguanosine-capped and polyadenylated small RNAs (CPA-sRNAs) [20] and sRNAs matching to repetitive elements, tRNA loci, rRNAs, protein coding genes, snRNAs and intergenic regions ([12]; this study). Moreover, several new classes of small RNAs have been described recently in fungi, including QDE-2-interacting siRNAs (qiRNAs), microRNA-like small RNAs (milRNAs) and Dicer-independent small interfering RNAs (disiRNAs) of *N. crassa*[21]. The existence of RNAi has been demonstrated in many fungal species including pathogenic species [22]–[24]. In *Mucor circinelloides*, production of two classes of small antisense RNAs requires a functional *Dcl-2* gene [25].

In the *M. oryzae* life cycle, transition from the biotrophic to the necrotrophic stage is likely induced by environmental cues that the fungus encounters during the pathogenic process [26]. Conditions encountered by the fungus during its growth in rice leaves, particularly during disease symptom expression, include nitrogen starvation, and this is regarded as one of the inductive cues for disease symptom expression during rice plant infections [26,27]. Starvation is also considered a key factor for inducing the germ tube tip to differentiate into an appressorium [28]. These and similar studies prompted us to examine whether post-transcriptional changes occur in *M. oryzae* during exposure to different physiological stressors, as well as during several invasive stages of pathogenic growth. Recent studies on genome-wide transcriptional changes demonstrate that *in vitro* conditions such as nitrogen and carbon deprivation are comparable to genome-wide changes that occur during invasive plant growth [29]. The role of post-transcriptional control during these stresses, however, has not yet been studied. We examined small RNA profiles of *M. oryzae* during stress conditions matching those of Mathioni et al. [29], namely, nitrogen starvation, carbon starvation, minimal media, and paraquat-induced oxidative stress. We also examined small RNA fungal profiles *in planta* at 72 and 96 hours post inoculation (hpi), as well as mock inoculated plants. Small RNAs from certain conditions showed association with specific genomic loci; for example we observed an abundance of sRNAs associated with retrotransposons during nitrogen starvation. We also identified a small subset of genes whose transcriptional down-regulation during several of the stress conditions showed association with high numbers of sRNAs. To further examine whether this represented a true link between transcriptional and post-transcriptional control, we generated four small RNA biosynthetic fungal mutants, and performed quantitative real-time RT-PCR on this down-regulated subset of genes. These results revealed a previously unknown role for the *Magnaporthe Dicer-Like1* (*MoDcl1*) gene.

## Results

### Small RNA profiling of *M. oryzae* stress libraries

We analyzed small RNA (sRNA) size profiles of libraries constructed from *M. oryzae* mycelial tissue, and from susceptible rice leaves infected with *M. oryzae* at two time-points. For the former, *M. oryzae* was subjected to the *in vitro* stresses of nitrogen starvation (NS), carbon starvation (CS), minimal media (MM), and oxidative-inducing conditions via paraquat (PQ). Mycelia were also grown on a rich, complete media (CM) for comparative purposes. Growth conditions for these *in vitro* stresses mimicked those of Mathioni et al. [29], and hereafter will be referred to as the “mycelial” libraries. In order to compare the *in vitro* stress sRNA profiles to small RNAs generated during *in planta* growth, we also isolated fungal-induced rice lesions at 72 and 96 hpi. These will be hereafter referred to as the “*in planta*” libraries. Small RNA libraries were generated and quality checked before performing deep sequencing using Illumina’s Genome Analyzer II (GA_II_) system. This resulted in 58,511,280 and 28,941,539 sequences from the mycelial and *in planta* libraries, respectively (Table 1). The sequences were aligned to the *M. oryzae* genome assembly version 6, downloaded from the Broad Institute (http://www.broadinstitute.org/). Total genome-matched reads (mapping to one or more sites in the *M. oryzae* genome) and distinct genome-matched reads were calculated. There were 34,652,258 and 2,414,112 total genome-matched reads, and 927,501 and 54,394 distinct genome-matched reads for mycelial and *in planta* libraries, respectively, (Table 1). For the *in planta* libraries, *M. oryzae* sequences were parsed out from rice sequences by alignment to the fungal genome. An informatics workflow describing this process is presented in Additional file 1: Figure S1, along with an example of the output from the pipeline as applied to a mycelial library.

**Table 1:** Description, total sequences, genome-matched reads and distinct reads for each sRNA library

**Code**	**Title**	**Total sequences**	**Genome-matched reads**^**a**^	**Distinct genome- matched reads**^**a**^
MgCM01	Mycelium, re-inoculated into complete medium for 16 hours	11,856,620	7,064,064	179,544
MgCS03	Mycelium, re-inoculated into carbon starved medium for 16 hours	12,601,478	8,115,777	333,810
MgMM04	Mycelium, re-inoculated into minimal medium for 16 hours	12,344,597	6,866,907	83,697
MgNS02	Mycelium, nitrogen-starved for 16 hours	11,532,596	7,310,147	211,570
MgPQ05	Mycelium, paraquat-treated for 24 hours	10,175,989	5,295,363	118,880
LMg0	Leaf, 0 hr control for Mg-treated libraries, Nipponbare	10,424,323	797,150	13,719
LMg72	Leaf, 72 hpi, Magnaporthe infected, Nipponbare	8,887,347	465,460	20,969
LMg96	Leaf, 96 hpi, Magnaporthe infected, Nipponbare	9,629,869	1,151,502	19,706

^a^ Does not include the data listed in the column "t/r/sn/snoRNA Matched Reads".

**Figure 1:**
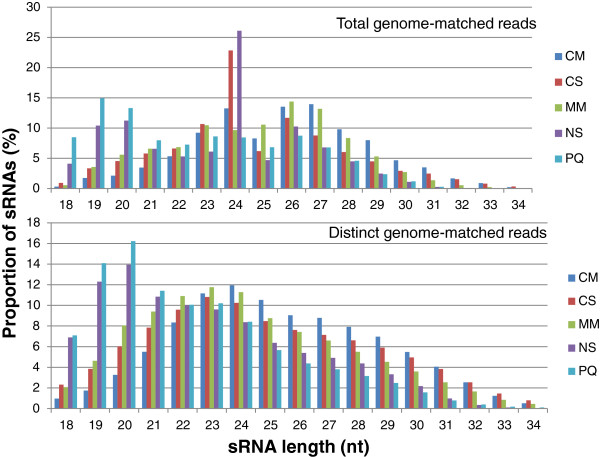
**Proportion of sRNAs in each size class for the environmental mycelial libraries.** Proportion of sRNAs for total genome-matched reads (top panel) and distinct-genome matched reads (bottom panel). Total genome-matched reads shows a peak at 24 nt for the starvation libraries, CS and NS, while the other libraries are roughly evenly distributed. Distinct genome-matched reads shows a relatively even distribution, except for NS and PQ, which have the highest peaks between 19–20 nt. CM = complete media; CS = carbon starved; MM = minimal media; NS = nitrogen starved; PQ = paraquat.

**Figure 2:**
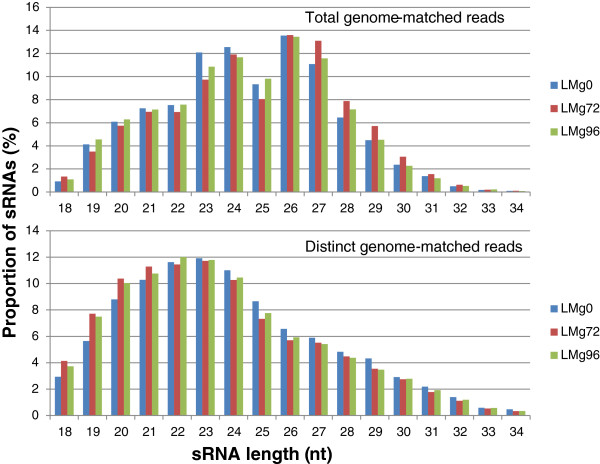
**Proportion of sRNAs in each size class for the *in planta *libraries.***M. oryzae* - specific reads were parsed out from the total reads by aligning them to the *M. oryzae* genome. Top panel: Proportion of total genome-matched reads shows a peak at 26 nt for the LMg72 and LMg96 libraries. Bottom panel: Proportion of distinct genome-matched reads shows peaks at 23 and 24-nt for the *in planta* libraries (bottom panel). LMg0 = mock inoculated; LMg72 = 72 hpi; LMg96 = 96 hpi.

The size distribution of total genome-matched reads for mycelial libraries were bimodal with peaks at 23/24 nucleotides (nt) and 26/27 nt, while distinct genome-matched reads showed a normal distribution curve with the peak at 23/24 nt (Figure 1). The abundance of 24 nt reads represented 16.77% of the total population. *In planta* libraries also had a similar pattern of abundance, but the major peak was at 26 nt followed by 24 nt for the total genome-matched reads and 22/23 nt for the distinct genome-matched reads (Figure 2). Almost 12% of the sRNAs are represented by the 24 nt class. Collectively, all mycelial libraries showed the majority of sRNA peaks at 24 followed by 26 nt, whereas *in planta* libraries showed the majority of sRNAs peaking at 26 followed by 24 nt.

### Cluster analysis of mycelial and *in planta* libraries

We performed a cluster analysis on each *M. oryzae* supercontig, based upon the proximity-based algorithm of Lu et al. [30] for a comparative analysis of the mycelial and *in planta* small RNA libraries. This clustering algorithm allowed us to determine the abundance of sRNAs and their distribution across the *M. oryzae* genome by dividing the genome into a series of 500 base pair (bp) windows (clusters) and calculating the abundance of sRNAs for each cluster. The clusters are defined by fixed coordinates, which allows comparative analyses of multiple libraries and the identification of differentially regulated clusters (DRCs) in the genome. In our study, DRCs represent regions of the genome with a sRNA abundance that differs by 10-fold or more in pairwise comparisons between the libraries. All mycelial libraries were compared to CM, which resulted in a total of 477 DRCs. We examined the total number of sRNA clusters for each library and identified clusters that map to genic (which includes UTRs, introns and exons) and intergenic regions, various forms of repeat regions and transposable element regions. Out of the 477 total sRNA clusters, 251 were from genic regions, 213 were from intergenic regions and 20 were from repeat regions and transposable element regions (Table 2). The CM vs. CS comparison revealed four DRCs, indicating carbon starvation had a minimal impact on the accumulation of sRNAs. NS vs. CM and PQ vs. CM had the greatest number of intergenic and genic-associated clusters. The genic and intergenic-associated clusters were 52 and 96 for NS vs. CM, and 179 and 69 for PQ vs. CM respectively. The NS vs. CM comparison resulted in the highest number (5) of sRNAs mapping to retrotransposons and transposons, versus other comparisons. Results from the *in planta* libraries showed that the majority of clusters associated with genes and intergenic regions, as opposed to other genomic regions, and this was similar to what was seen in the CM vs. PQ comparison. Overall, the mycelial libraries showed more small RNAs mapping to transposons and retrotransposons, compared to the *in planta* libraries; for the latter libraries, with the exception of two clusters mapping to retrotransposons, almost everything else associated with genic regions (Table 2).

**Table 2:** Cluster analysis of all small RNA libraries compared between two conditions

**Libraries compared**	**Total clusters**^**a**^	**No. of genes associated**	**RTNs**^**b**^	**TNs**^**c**^	**Inverted repeats**	**Tandem repeats**	**Other repeats**	**Intergenic regions**
Mycelial libraries								
CM vs CS	4	2	0	0	0	0	0	2
CM vs MM	37	12	1	0	0	0	0	24
CS vs MM	31	6	0	0	2	0	0	23
NS vs CM	154	52	4	1	3	2	0	96
CM vs PQ	251	179	2	0	1	3	1	69
Infected Rice leaf libraries								
LMg0 vs 72	281	247	0	0	0	1	3	38
LMg0 vs 96	232	192	2	0	0	2	3	39
LMg72 vs 96	188	164	0	0	0	2	2	29

^a^ Several of these clusters associate with more than one genomic element.

^b^ Retrotransposons.

^c^ Transposons.

### sRNA associate with different genomic regions, depending upon the fungal stress

In total, taking data from all the libraries together, the majority of sRNAs were found to be associated with intergenic regions (54%), followed by repeat-containing regions (41%; Table 3). On the other hand, comparisons across libraries resulted in more DRCs associated with genes (Table 3). Although more total sRNAs mapped to intergenic regions compared to protein-coding regions, protein-coding regions contained a greater number of distinct sRNAs (Table 2). This difference may be explained by the presence of repeats which have not yet been annotated, and which were not identified by the RepeatMasker repeat identification program ([31]; http://www.repeatmasker.org). Though approximately 50% of the genome codes for proteins and 7% is occupied by repeats [6], our sRNA data suggests only 4% of the total sRNAs sequenced matched to protein coding regions.

**Table 3:** Total, and distinct genome-matched sRNAs among all libraries for different classes of genomic elements

**Class**	**Total sRNAs**	**Distinct sRNAs**
Protein-coding genes	2,084,221	368,507
Antisense to protein-coding genes	635,227	71,846
Intergenic regions	38,026,665	231,988
Repeats	28,238,241	73,293
• DNA/TcMar-Pogo	7,353	291
• LINE/Tad1	68,533	2,983
• Low complexity	16	9
• LTR/Gypsy	3,126,380	37,857
• rRNA	25,039,488	31,775
• Simple repeat	1,888	407
Mitochondrial sequences	418,691	9,310
Noncoding RNAs (tRNAs or rRNAs)	15,297	4,281
Antisense to noncoding RNAs	2,379	762

To determine whether our libraries identified repeat-associated siRNAs (known as rasiRNAs), we analyzed six different classes of repeats, and found that the majority of the sRNAs emanated from rRNA loci (89%) followed by retrotransposons (LTR/Gypsy and LINE/Tad1; 11%). Small RNAs that matched to transposons (DNA/TcMar-Pogo), simple repeats, and low complexity repeats occupied only a small fraction of the total sRNAs. Though rRNA-associated sRNAs are more abundant compared to retrotransposons-associated sRNAs, the distinct reads associated with the former were less (43%) compared to the latter (52%). The majority of sRNAs matching to protein-coding regions were in a sense orientation (77%; Table 3), and a similar phenomenon was observed for non-coding RNAs (tRNAs or rRNAs; 87%).

To further characterize the sRNAs associated with retrotransposons, representative loci from major repeat classes in *M. oryzae* such as LTR/Gypsy and LINE/Tad1 retrotransposons were analyzed for their size distribution, changes under different physiological conditions, strand specificity and 5’ nt identity. Strand orientation of sRNAs was also included in our analysis because abundant antisense siRNAs are known to be associated with repeats [32] and are affected by the loss of RNAi machinery [5]. Also, studying strand specificity is important because sRNAs originating from overlapping gene pairs or natural cis-antisense gene pairs have been shown to play an important role in salt tolerance in Arabidopsis [33] and gene regulation in mouse oocytes [34]. LTR/Gypsy retrotransposons showed major peaks at 20–23 nt and abundant sRNAs in the antisense orientation (Additional file 2: Figure S2A and B). These sRNAs differentially accumulated under different physiological stress conditions; for example, one class of LTR/GYPSY retrotransposon called GYMAG2 showed a spike in sRNA accumulation during nitrogen and oxidative stress conditions that was roughly 28% and 50% higher than any other condition (Additional file 2: Figure S2D). Analysis of the next most abundant major repeat class of retrotransposons, LINE/Tad1, revealed that its major peak for sRNAs based on abundance, was at 20 nt followed by 23/24 nt (Additional file 3: Figure S3A). Similar to the LTR/Gypsy retrotransposon class, sRNAs matching to LINE/Tad1 repeats also showed differential accumulation in various physiological stress conditions, but unlike the LTR class, LINE/Tad1 sRNAs did not show a spike under any one particular condition (Additional file 3: Figure S3C). Unlike LTR/Gypsy, sRNAs matching to LINE/Tad1 did not show abundant sRNAs in the antisense orientation (Additional file 3: Figure S3B).

We observed that many of the annotated tRNAs and 5S rRNAs represent a significant portion of intergenic region -associated sRNAs. In order to study these genomic features, we selected random loci of each feature and analyzed their size distribution. The results showed a bimodal distribution for sRNAs originating from a majority of the rRNA loci, with size peaks at 20 and 23/24 nt (Additional file 4: Figure S4). Analysis of random tRNA loci revealed the presence of varied size distribution and increased sRNAs under the PQ condition (Additional file 4: Figure S4). We also observed strand bias for sRNAs originating from 5S rRNA and tRNA. All analyzed 5S rRNA and tRNA loci showed sRNAs on the sense strand (data not shown). sRNAs originating from mitochondrial sequences occupied only a small proportion, comprising 0.6% and 1.2% of total and distinct genome-matched reads, respectively (Table 3).

Stress conditions altered the proportion of individual classes of sRNAs associated with different genomic features (Additional file 5: Figure S5A). The PQ library revealed a notable reduction in both total and distinct abundance sRNAs matching to protein coding regions, compared to all the other libraries. Distinct abundance for the PQ library also showed an increase in sRNAs associated with intergenic regions, and with repeats. Another prominent difference was observed in the CS library; there was an increase in sRNAs associated with LTR Gypsy transposons, and a decrease in sRNAs associated with rRNA repeats (Additional file 5: Figure S5C). An increase in LTR retrotransposons was also observed during infection of rice plants with *M. oryzae* (Additional file 5: Figure S5D).

### 5’ nucleotide preference

**Figure 3:**
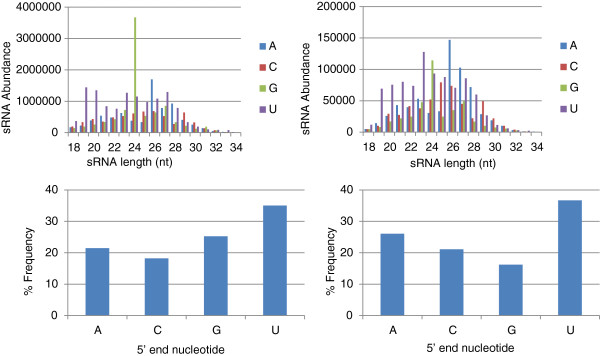
**5’ nucleotide preference for sRNAs in *M. oryzae *mycelial libraries (left panels) and *in planta *libraries (right panels).** In the mycelial libraries, there was a strong preference shown for G, while in the *in planta* libraries, there was a preference shown for A followed by U.

Nucleotide preference in the 5’ position is the determining factor in plants for binding of sRNAs to specific AGO proteins [35]. While fungi do not appear to share this exact characteristic, preference for particular 5’ nucleotides have been observed [12,21,36,37]. In *M. oryzae*, analysis of different libraries for their 5’ nt preference revealed that sRNAs starting with U were abundant in both mycelial and *in planta* libraries, and there was a suppression for C in mycelial libraries and for G in *in planta* libraries (Figure 3). The 5’ U preference was maintained in individual mycelial libraries – CM, MM, NS and PQ, but the CS library contained more sRNAs starting with G, closely followed by U. The suppression of C at the 5’ position was also maintained in most of the mycelial libraries except for MM and PQ (data not shown). The 5’ end nucleotide preference for the LTR class was U, and for LINE/Tad1 was both A and U (data not shown). The general pattern of abundant U and less G for sRNAs from *in planta* libraries was reflected in the LMg0 and LMg96, while LMg72 library had abundant A and less G (data not shown).

### Correlation between transcriptional and post-transcriptional regulation under stress conditions

**Figure 4:**
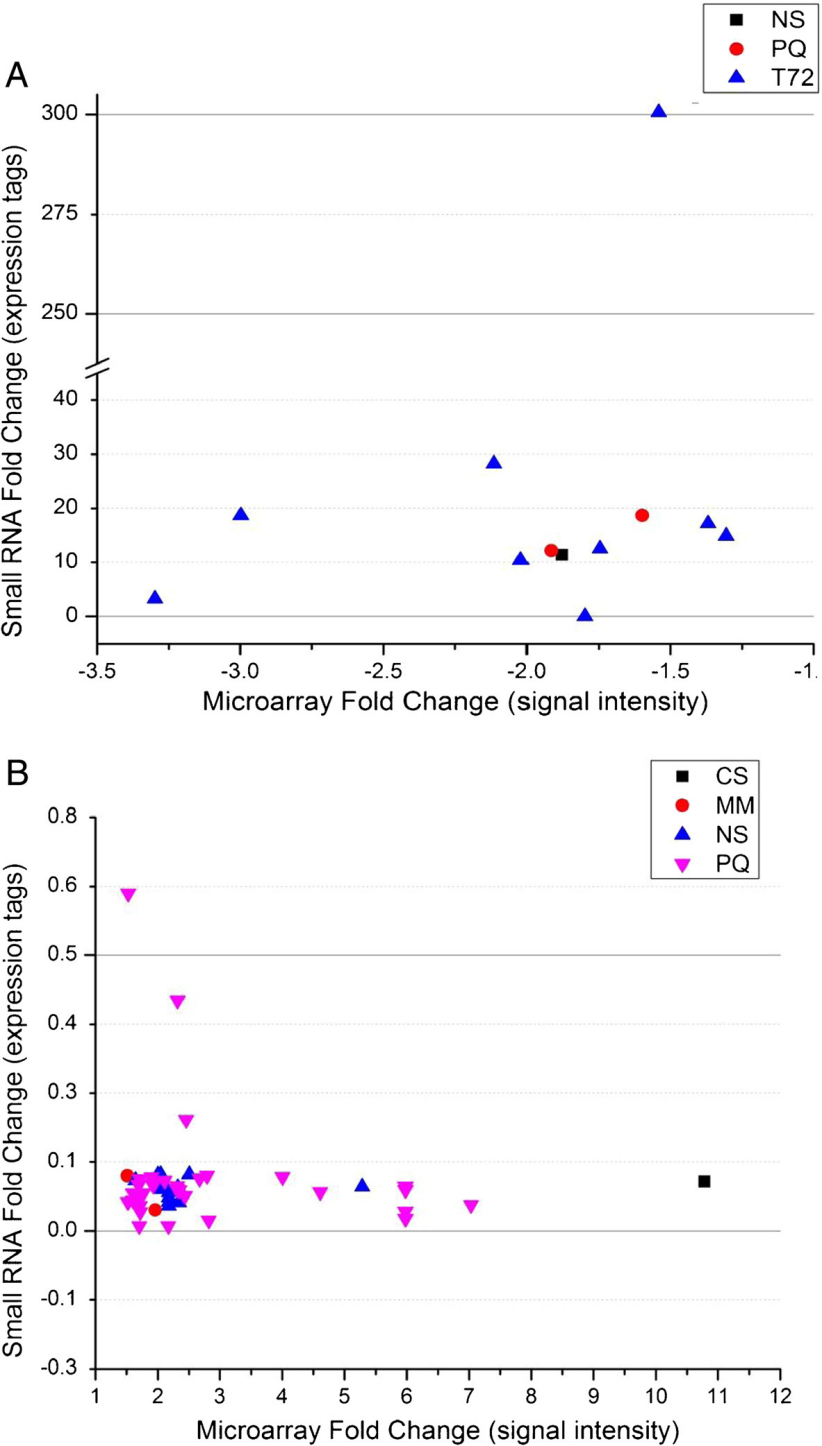
**Association between transcriptional control of genes within the different environmental conditions and post-transcriptional control of the same set of genes under the same conditions.** Genes from three different environmental conditions are negatively regulated in the microarray study for that condition, while they showed an abundance of small RNAs associated with the gene for that condition compared to the complete media (CM) control (**A**); Genes from four different environmental conditions were induced in the microarray for that condition, while they showed a low amount of sRNAs associated with the gene compared to the CM control (**B**). CS = carbon starved; MM = minimal media; NS = nitrogen starved; PQ = paraquat; T72 = Infected rice leaf at 72hpi.

We hypothesized that transcriptional down-regulation of genes may correlate with a plethora of sRNAs accumulating either around that gene in the intergenic spaces, or within the gene itself. In order to gain a genome-wide perspective on how transcriptional and post-transcriptional gene regulation might be related, we compared our sRNA data to transcriptional genome data generated from *M. oryzae* under the same “stress” conditions as Mathioni et al. [29] in their microarray study. From this dataset, we identified 98 genes associated with clusters that showed differential accumulation of sRNAs (≥ 10-fold compared to CM control), 28 and 9 of which showed a negative correlation with microarray data under PQ and NS conditions, respectively (Additional file 6: Figure S6; Additional file 7: Table S1; Additional file 8: Table S2; Figure 4). Further analysis of four of these genes showed that they have sRNAs originating from exons or introns in either the sense or antisense orientations (Table 4). Of these, only MGG_16711.7 (earlier annotated as MGG_05869.6) coding for a hypothetical protein containing an importin-beta N-terminal domain, had many sRNAs originating from its intron.

**Table 4:** Features of sRNAs associated with transcriptionally down-regulated genes under different physiological stress conditions

**Gene ID/Description**	**Condition**	**sRNA**^**a**^	**Microarray**^**b**^	**Signature**	**Origin**
MGG_01439.7 inorganic phosphate transporter PHO84	PQ	95.00	-5.04	TGGGCTCGAGAGCAAGGCG	Exon sense
MGG_04470.7 nucleolar complex protein 14	NS	11.43	-1.88	TTGGCTGTGAATTCGGCG ^c^	Exon antisense
				TTGGCTGTGAATTCGGCGT	Exon antisense
				TGGCTGTGAATTCGGCGT	Exon antisense
				CGAGTCTGATGCTGAGAGCTCAGGCA	Exon sense
				CGGAGAGGACGGAGAGGAGCTCC	Exon sense
MGG_01439.7 inorganic phosphate transporter PHO84	NS	89.5	-2.71	GGGAAGGATGGACAGGGG	Exon sense
MGG_16711.7 ^C^ Hypothetical protein	PQ	12.18	-1.92	AGCGTTTCTACTTTCTGATCACA	Intron sense
				AAGCGTTTCTACTTTCTGATCACA	Intron sense
				AAGCGTTTCTACTTTCTGATC	Intron sense
				CGGGTGCTGAGAAAGCGTT	Intron sense
				GCGGGTGCTGAGAAAGCGTTTCT	Intron sense
				GCGGGTGCTGAGAAAGCGTTT	Intron sense
				GCGGGTGCTGAGAAAGCGTT	Intron sense
				GCGGGTGCTGAGAAAGCGT	Intron sense
				GCGGGTGCTGAGAAAGCG	Intron sense
				TGCGGGTGCTGAGAAAGCGTT	Intron sense
				TGCGGGTGCTGAGAAAGCGT	Intron sense
				TGCGGGTGCTGAGAAAGCG	Intron sense
				TTGCGGGTGCTGAGAAAGCGTTTCT	Intron sense
				TTGCGGGTGCTGAGAAAGCGTT	Intron sense
				AATTGCGGGTGCTGAGAAAGCGTT	Intron sense
				GTGAAGTGATGAATACAATGCGT	Intron sense
				GTGAAGTGATGAATACAATGCG	Intron sense

^a^ Ratio of sRNA between particular physiological condition and CM library based on cluster data.

^b^ Expression of genes at particular physiological condition compare to CM using microarray.

^c^ This gene is selected to replace MGG_05869.6 of the annotation version6.

For the *in planta* libraries, we were most interested in sRNAs that were exclusively present at 72 hpi and/or 96 hpi (Additional file 9: Figure S7), indicating that these fungal sRNAs might be involved in invasive growth. In order to determine whether any of these sRNAs were negatively correlated with genes from the microarray study as described above, we first selected genes associated with sRNA clusters from the comparisons of LMg0 vs. LMg72, LMg0 vs. LMg96 and LMg72 vs. LMg96 that showed a ≥ 10-fold difference. Next, their corresponding loci from the CM vs. LMg72 data were compared to the microarray study [29]. This resulted in 12 genes that were both down-regulated in the microarray, associated with large quantities of sRNAs (Table 5). For each gene, abundant sRNAs present in LMg72 and/or LMg96 were analyzed for their position, and checked for their absence in non-inoculated rice sRNA libraries (http://mpss.udel.edu/rice_sRNA), indicating that they were originating from the fungal, and not the rice, genome. The resultant sRNAs were largely found to be associated with exons, 5’ UTR or 3’ UTRs in a sense orientation (Table 5). Interestingly, one sRNA matching to MGG_08843 coding for the magnesium transporter, ALR2, was abundant at 72 hpi and present in an antisense orientation to the 5’UTR; Mathioni et al. [29] demonstrated down-regulation of this gene during carbon starvation, as well as *in planta* growth in rice and barley. Another gene includes the well-characterized transcription factor, *ACE1* (MGG_04428.7); this gene is of interest, as it is known to be tightly regulated and involved in appressorial formation [38].

**Table 5:** Features of sRNAs associated with fungal genes down-regulated during infection

**Gene ID/Description**	**sRNA**^**a**^	**Microarray**^**b**^	**Signature**	**Chromosome**	**Origin**	**LMg72**^**c**^	**LMg96 **^**c**^
MGG_02390.7 conserved hypothetical protein	14.91	-1.31	GCAGGCAGAGGAACACTGAAGCA	1	3’UTR sense	287	0
			CTGCAGGGCTGTCTTCTGATGGA		Exon sense	0	162
			CTGCAGGGCTGTCTTCTGATGG		Exon sense	0	137
			CGGCGACTTAGCTGCCTCTGAACCCGGCTACCAT		5’UTR sense	0	150
MGG_06609.7 acetyl-CoA hydrolase	17.18	-1.37	CAAGGAGAGGATTCTGTTGCGATCGCAGT	4	Exon sense	790	0
MGG_09965.7 conserved hypothetical protein	0.00	-1.80	GAACAGGGCTGGCTTGCCTGACAAC	4	5’UTR sense	503	0
MGG_08843.7 magnesium transporter ALR2	36.75	-3.30	GATGACTTGGAAGTATGAAGCCAGCGTGATGG	2	5’UTR antisense	395	0
			AACACGTCGGGAACTCGGGCTA		5’UTR sense	215	0
MGG_04696.7			CGGTTGGACAGAGTATTCGGCA	2	3’UTR sense	0	175
			TCGGTTGGACAGAGTATTCGGCAGTTCGA		3’UTR sense	0	12
			TCGGTTGGACAGAGTATTCGGCAG		3’UTR sense	36	0
			ATCGGTTGGACAGAGTATTCGGCAGT		3’UTR sense	0	100
			TGAAGAAGGTTCTCTGCATGGGT		Exon sense	108	0
			TCCAGCTGAAGAAGGTTCTCTGCATGG		Exon sense	0	100
			GGCTGACGACTTGAAGAAGCTGAA		Exon sense	251	225
			CGGCTGACGACTTGAAGAAGCTGAA		Exon sense	323	262
			CATGTCGGCTGACGACTTGAAGAAGCTGA		Exon sense	0	137
			CGAGACCGTCGAGCTCCAGATCGGCCT		Exon sense	0	125
			CTCGAGACCGTCGAGCTCCAGATCGG		Exon sense	0	137
MGG_01991.7 betaine aldehyde dehydrogenase	12.55	-1.75	CAGTGGTCTCGGCACTGAGAACGGT	1	Exon sense	180	0
			TCAAGTGGTTTCGGTACTACGCAGCT		Exon sense	36	0
MGG_06868.7 acetolactate synthase catalytic subunit	10.42	-2.02	GTGGAAGGAGAAGTGGCCTCTGTCACA	1	Exon sense	287	0
			CAACATGACTCTGACAGAGCTTTCGACGGCG		Exon sense	467	0
			ACATGACTCTGACAGAGCTTTCGAC		Exon sense	0	162
			AGCCTGACGATGTCGTTGATGCTC		Exon sense	359	0
			AGCCTGACGATGTCGTTGATGCT		Exon sense	610	0
			TTGTCGGAAGGCGGCGTTGAACTTC		Exon sense	0	87
MGG_04428.7 zinc finger transcription factor ace1	28.29	-2.11	CATGGCTCGCCGCAAGAAGAACG	2	Exon sense	180	0
			AAGGCCATCTCACTGGCCACTG		Exon sense	0	37
MGG_01596.7 DNA damage response protein kinase DUN1	18.67	-3.00	CAGGCAAGGGCAAGGAACACT	2	Exon sense	251	0
			AAGGAACACTGAACCGTGGAAC		Exon sense	215	0
MGG_04994.7			CGACTCTGACGACGAGGATGGAAC	3	Exon sense	0	237
			CAGATCTCCCTGACTGAGAACTGGCT		Exon sense	287	0
			CGGTTTCGGTTGGTTCTCTGGCGGCC		Exon sense	0	75
			TTCGGTTGGTTCTCTGGCGGCCTCGGC		Exon sense	36	0
			GTTGGTTCTCTGGCGGCCTCGGCGAG		Exon sense	0	150
MGG_09395.7			has Tandem repeats on it	6			
MGG_06714.7			CAAGGACAAGTGGGTGTTGAACGACAGC	1	Exon sense	539	0

^a^ Ratio of sRNA between rice library and CM library based on cluster data.

^b^ Expression of genes at 72hpi in rice compared to CM using microarray data [29].

^c^ Abundance of signature.

**Figure 5:**
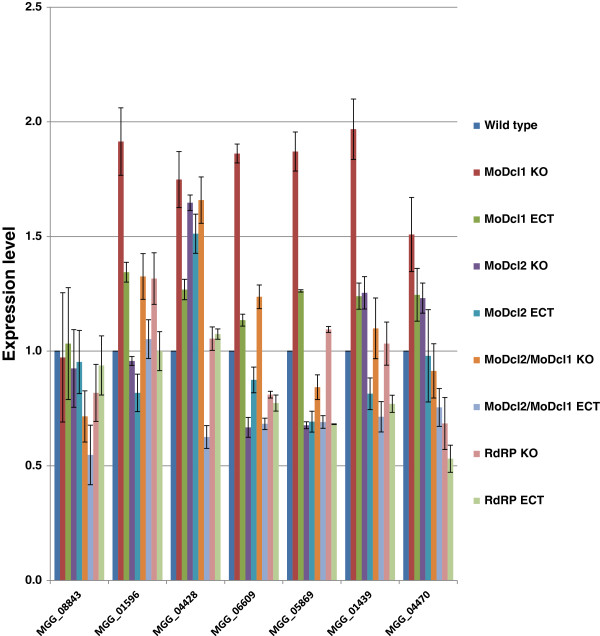
**Expression of selected genes in different mutant backgrounds.** In order to understand the regulation of genes with a negative correlation between sRNA levels and microarray expression, seven genes were selected and analyzed in wild type and in RNAi mutant backgrounds using real time qRT-PCR; expression levels were calculated using 2^-ΔΔCT^ method. MoDcl1: Dicer1; MoDcl2: Dicer2; RdRP: RNA-dependent RNA polymerase (MGG_13453); MoDcl2/MoDcl1: Dicer2 knock-out in a Dicer1 knock-out background (the reciprocal was also performed and showed similar results); KO: knock-out; ECT: ectopic. MGG numbers correspond to the following genes: MGG_08843 = magnesium transporter ALR2; MGG_01596 = damage-response protein DUN1; MGG_04428 = ACE1; MGG_06609 = acetyl-CoA hydrolase; MGG_05869 = importin domain-containing; MGG_01439 = inorganic phosphate transporter; MGG_04470 = nucleolar complex protein 14.

In order to confirm whether these genes might truly be under post-transcriptional regulation, we generated four sRNA biosynthetic mutants, and utilized real-time qRT-PCR to determine the expression of seven genes identified from the above analysis, namely MGG_08843.6, MGG_01596.6, MGG_04428.6, MGG_06609.6, MGG_05869.6, MGG_01439.6 and MGG_04470.6. Mutants tested included gene replacement deletions in the following loci: Dicer 1 and Dicer 2 (MGG_01541 and MGG_12357, previously generated and characterized by Kadotani et al. 2002, and hereafter referred to as *MoDcl-1* and *MoDcl-2*), one RNA-dependent RNA Polymerase (RdRP MGG_13453; hereafter referred to as *RdRP*) and a double-knockout mutant in both Dicers (see Additional file 10: Figure S8 for deletion construct details, confirmation of deletions and Southern hybridization, revealing one disruption cassette per mutant line). We hypothesized that suppression of gene expression would be lifted if the genes for generating sRNAs in the fungus were no longer present, and sRNAs were no longer made. Six of the seven genes analyzed showed 1.5 to 1.9-fold increased expression over the wild type in the *MoDcl1* mutant. The MGG_01596 gene, encoding a DNA damage response protein *DUN1*, increased in expression in the ∆*modcl1, modcl2/modcl1* and in the ∆*mordrp-1* and was reduced in the ∆*modcl2* mutant backgrounds. The MGG_04428 gene encoding the transcription factor *ACE1* was increased ~1.7-fold in the ∆*modcl1* background (Figure 5).

## Discussion

Only a small fraction of RNA species (2-3%) are translated into proteins in higher organisms, while the remainder includes non-coding yet functional species such as small RNAs [39,40]. Small RNAs in eukaryotes have some well-established roles in modulating gene expression at the transcriptional and post-transcriptional levels, as well as in shaping the organization and modification of chromatin [41]. There is a robust body of knowledge on sRNAs in the model fungus *Neurospora crassa*, and in the budding yeast ([5]; reviewed in [10,42]). Recently, several detailed studies on sRNAs have emerged in fungal pathogens, including *M. oryzae* amongst others [11,12,20,21,37]. The current study presented here, adds to this growing information by analyzing sRNAs under various physiological stress conditions and during plant infection. Understanding the epigenetic response of plant pathogens to different environmental conditions may elucidate how these pathogens so successfully adapt to new climates and/or new hosts.

Our results demonstrate that the majority of the sRNAs originate from intergenic regions followed by repeats, protein coding regions and mitochondrial regions, when sRNAs from all libraries were taken together. Our results are supported by those of Nunes et al. [12], who reported abundant sRNAs matching to intergenic regions, transposable elements, rRNA, tRNA, and protein-coding genes, from mycelial and appressorial libraries. Transposons account for about 7% of the *M. oryzae* genome (including 5.4% contribution by retrotransposon; [6]); our data suggests that only 4.5% of the total sRNA population matches to transposons (11% of the repeats population), which indicates RNAi may not act as the only mechanism to suppress transposons in *M. oryzae*. This observation is further supported by the hypothesis of Murata et al. [43], which supports the operation of a transposon- or repeat-silencing mechanism distinct from both transcriptional gene silencing and siRNA-mediated gene silencing.

After spore germination and appressorium formation, *M. oryzae* generates a penetration peg that enters into the plant’s epidermal cell. Primary infection hyphae colonize this cell followed by formation of bulbous secondary hyphae and spreading to adjacent epidermal cells (the biotrophic phase; [44]). At about 72 hpi, secondary hyphae spread into mesophyll cells and at this time approximately 10% of total biomass is fungal [45], which could account for the reduced amount of total and distinct genome matched reads in *in planta* libraries, compared to the mycelial libraries. Under favorable disease conditions, visible lesions are formed by 96 hpi [45]. The drastic changes undergone by the pathogen during invasive, later stage growth are reflected in the increased number of genes showing differential abundance for sRNAs during 72 and 96 hpi, as determined by the cluster analysis (Table 2). During these later invasive stages, the fungus is believed to be coping with nutrient-limited environments [27,29]. In the *in planta* libraries, we observed 562 distinct reads associated with LMg72 compared to LMg96 after the removal of LMg0, which represent rice-only reads (Additional file 9: Figure S7). Furthermore, out of the ~8000-9000 sRNA reads found in both the LMg72 and LMg96 libraries, only 2132 overlapped, indicating that many unique small RNAs are generated during these invasive, later growth stages. We took advantage of previous transcriptional data generated from our lab in 2011 to determine whether clusters with sRNAs have a negative correlation with significantly differentially expressed genes in the microarray study. Ninety-eight such genes from the microarray study associated with sRNAs and 37.7% of these showed a negative correlation indicating that these particular genes may be regulated epigenetically. The remaining ~60% of the microarray genes showed a positive correlation with the sRNA data. While these are relatively low numbers from a genome-wide perspective, the microarray data was generated from a very early version of the *M. oryzae* genome annotation, which has changed considerably in the last four years; furthermore, the 98 genes from the microarray study were based upon highly stringent criteria used both in this study, as well as the study from 2011, limiting the number of such genes we can identify.

### Small RNAs differentially associate with retrotransposons, in a stress-dependent manner

Living organisms adapt to the environmental stress conditions by undergoing several phenotypic, physiologic and molecular changes, which requires precise regulation at the transcriptional, post-transcriptional and translational levels. These changes include but are not limited to activation and repression of several genes [46], signaling cascades [47], changes in molecular chaperones [48], activation of transposable elements [49,50] and chromatin changes and epigenetic modifications [51]. Several nutrient stress responsive sRNAs, have been identified in plants [52,53], bacteria [54] and *Caenorhabditis elegans*[55].

In pathogenic fungal species, efficient pathogen nutrition is essential for successful colonization and fungal fitness [56]. Transcriptome studies on *M. oryzae* and many other fungi have demonstrated that nutrient acquisition is a tightly regulated process, involving genes that function in amino acid metabolism and uptake, global nitrogen regulation and pathogenicity [27,57]–[59]. In the present study, we found that repeat-associated sRNAs generally and LTR-retrotransposons specifically were induced by oxidative stress and nitrogen starvation, respectively (Additional file 5: Figure S5). This observation may be similar to Arabidopsis roots undergoing phosphate starvation, which induces a high level of DICER-LIKE1-dependent sRNAs derived from the long terminal repeat of a retrotransposon and a group of 19 nt sRNAs that correspond to the 5’ end of tRNA [60]. In rice, some repeat-derived sRNAs are down-regulated by abscisic acid and abiotic stresses accompanied by an increased accumulation of their precursors [61].

In *M. oryzae*, MAGGY retrotransposons are activated by heat shock and oxidative stress along with up-regulation of MAGGY RNA [62] and an EST matching to a non-LTR-retrotransposon was identified in an *M. oryzae-*infected rice leaf library [63]. Similarly, our results revealed a spike in sRNAs associated with an LTR class during oxidative stress. Several ESTs matching to LTR-retrotransposons have also been identified in the *in planta* stage of the wheat fungal pathogen, *Mycosphaerella graminicola*, and are less abundant compared to their mycelial stage [64]. Similarly, our data revealed that distinct sRNAs reads matching to repeats in *in planta* growth were ~10% higher than in the mycelial libraries (Additional file 5: Figure S5A and B). Reactivation of transposable elements during stress conditions have been found in many organisms from fission yeast to plants and animals (reviewed in [65]). Recently, a widely-studied tobacco transposon called *TNT1* was found to be reactivated during infection of tobacco [66]. While we did see an increase in sRNAs associated with LTR/Gypsy retrotransposons during nitrogen starvation compared to the non-stressed condition, overall, there was not a striking difference in these types of associations in the case of the LINE/Tad1 class of retrotransposon, where sRNA abundance was not markedly different in the stress versus non-stressed conditions. Together, our data and the wealth of information on transposon-associated small RNAs in other organisms suggest that retrotransposons might be suppressed via epigenetic mechanisms. Additional testing will be required to determine what underlies the increase in sRNAs associated with LTR elements during nitrogen starvation.

Interestingly, we noted an abundance of sRNAs on the antisense strands of LTR/Gypsy elements, while there was a roughly equal distribution of them on either strand of the LINE/Tad1 elements. This was different from the recent study by Nunes et al. [12] on sRNA species in *M. oryzae*, which showed a more equal distribution of siRNAs on both LTR-associated strands, and may be attributable to the differing conditions used in each study and differing sequencing depth. Association of small RNAs with one strand or the other has been well-studied in Drosophila, for example, antisense siRNAs are abundant for repeat regions [32]. In general, transcription of transposons is activated by stress conditions; for example, carbon or nitrogen stresses activate the transcription of Drifter, a novel low copy transposon in *Fusarium oxysporum*. Some of the transposons are transcribed in both directions [67] and environmental stress-regulated antisense transcription of Ty1 LTR-retrotransposons is observed in *S. cerevisiae*[68].

### Derivation of the sRNAs in *M. oryzae*: antisense and 5’ nucleotide position

Around 25% of the tags that matched to genes are in antisense orientation (Table 3). Interestingly, one-fifth of the annotated genes of this fungus encode both sense and antisense transcripts [69]. The sources of the dsRNAs for Dicer processing include inverted repeats, bidirectional transcription and antisense transcripts from various loci [34]. There is a possibility that overlapping transcripts in an antisense orientation produced from a pair of neighboring genes on opposite DNA strands (convergent overlapping gene pairs or natural cis-antisense gene pairs) form dsRNAs, which may be further processed into sRNAs [33,34]. Recently, antisense transcripts were also found in other fungal species like the ectomycorrizal ascomycete *Tuber melanosporum*[70] and basidiomycete mushroom *Schizophyllus commune*[71]. It is also proven that siRNAs matching to mRNAs in *S. castellii* and in the RNAi reconstituted strain of *S. cerevisiae* were indeed produced from dsRNA formed by antisense and overlapping transcription [5,72]. Even in *M. circinelloides*, the majority of the exonic-siRNAS (ex-siRNAs) are antisense to mRNA [37]. Together, these results suggest that at least part of the siRNAs that matched to genes are produced from dsRNA formed by overlapping sense and antisense transcripts in *M. oryzae*. We observed that both 5S rRNA and tRNA loci were producing more of sense strand specific sRNAs whereas pseudo-tRNA loci have both sense and antisense sRNAs (data not shown). It has been shown that pseudo-tRNA of *Bacillus cereus* is detected to have a function outside protein synthesis [73]. Moreover, a subset of pseudogenes generates endo-siRNAs from double-stranded RNAs or directly in mouse oocytes [74]. The same scenario may exist in *M. oryzae*, because around 40% of pseudo-tRNAs are present either within a gene or opposite strand to it.

In the RNAi pathway, the Dicer-cleaved sRNAs are bound by Argonautes, and based on their 3’ complementarity Argonautes exert homology dependent transcriptional cleavage or post-transcriptional silencing [75]. The binding of sRNAs to AGO is mainly dependent on structural features of precursor duplexes in animals [76,77] whereas in plants, it is determined by the 5’ nt of the sRNA. In *Arabidopsis* AGO2 and AGO4 preferentially recruit sRNAs with 5’ A, likewise AGO1 with U and AGO5 with C [35]. Abundant U at the 5’ end observed in all the mycelial libraries in our study may be biologically significant (Figure 3), since this kind of preference was observed in other organisms including the single cell green alga *Chlamydomonas*[78]. Importantly, a preference for U (T) and suppression of C was observed in *M. oryzae* in a recent study, which our data supports [12]. In the fungal kingdom, 5’ preference for U is observed in qde-2 (Argonaute) associated sRNAs and milRNAs, and disiRNAs of *N. crassa*[21]; ago-1 associated sRNAs of *S. pombe*[36]; sRNAs of *S. castelleii* and *K. polysporus*[5]; and Ex-siRNAs of *Mucor circinelloides*[37].

### A role for Dicer1 (MoDcl1) in *M. oryzae*?

Though Dicer-like (DCL) proteins are evolutionarily conserved in eukaryotes, their numbers vary among organisms. In the multiple DCL protein-containing organisms such as Drosophila and *A. thaliana*, they function in distinct RNA silencing pathways with overlapping specificity [79]. Both *M. oryzae* and its close relative *N. crassa* encode two Dicers. However, MoDcl2 in *M. oryzae* functions in siRNA production under normal conditions, whereas *N. crassa* Dicers, orthologs of the *M. oryzae* Dicers, act redundantly. While it is likely that MoDcl1 does not have the ability to render or exert a silencing role under normal conditions either triggered by hairpin RNA-expressing transgenes or endogenous repetitive sequences, it is capable of producing siRNAs when overexpressed in an *MoDcl2* deletion background. Moreover, its mRNA levels were up-regulated by 10- to 15- fold during the sexual stage. It has been proven that both *MoDcl1* and *MoDcl2* define functionally diversified RNAi pathways which may have arisen from transcriptional control and protein specialization [80].

In *M. oryzae*, deletion of *MoDcl2* leads to increased abundance of MAGGY mRNA when it is introduced into the native genome [43]. With these reports in mind, it was expected that deletion of *MoDcl2* in our experiments would increase the transcript abundance of genes which are known to possess more sRNAs in one or more libraries, in comparison with mycelia grown in complete media (control condition). Conversely, our results showed there is an overall reduction in transcript levels of these genes in the *Δmodcl2* mutant and an increased level in the *Δmodcl1* mutant. Interestingly, the known virulence gene *ACE1* was among the few genes that fit this profile. *ACE1* is tightly regulated at the transcriptional level; transcripts are only found in the penetration peg of the fungus, a specialized hypha that allows ingress through the surface of the leaf and into the plant cell [81]. We postulate that strong control of this gene might be governed at the post-transcriptional level. Further studies will reveal whether these and the other genes tested show the same profile when the biosynthetic mutants are subjected to the environmental stress conditions, or during pathogenicity. Several studies have shown that MoDcl2 is exclusively involved in siRNA production for transgenes and active transposons in *M. oryzae*, while less active transposons are not silenced by this same mechanism [43,80,82]. Moreover, MoDcl1 is also involved in silencing when it is over-expressed in a *Δmodcl2* mutant background. Together, these findings along with results presented here suggest the hypothesis that MoDcl1, at least in *M. oryzae*, might define the existence of a MoDcl2-independent RNAi mechanism. The recent discovery of Argonaute associated Dicer independent siRNAs (disiRNAs) in *N. crassa*, the first discovered Dicer-independent siRNAs in the fungal kingdom, supports this hypothesis. Surprisingly, in *M. circinelloides*, Dicer2 plays a critical role in transgene-induced silencing [25], but redundancy for the two Dicer genes was observed for production of endogenous sRNAs with DCL2 being the primary protein responsible for producing the majority of endogenous sRNAs [37]. Taken together, these studies suggest that *M. oryzae* presumably has similar redundant Dicer-based mechanisms for the production of sRNAs.

## Conclusion

In conclusion, various stress-inducing and *in planta* conditions alter the sRNA profile of *M. oryzae*, with the main effect exerted on loci associated with protein coding regions. A subset of these genes showed an association with the transcriptome data, as well as a differential accumulation of their transcripts between the two *Dicer*-deficient mutant backgrounds. Stress conditions repressed the sRNAs associated with protein coding regions, and marginally increased the intergenic- and repeat- associated sRNAs. Considering our results and previous reports, questions of Dicer-independent mechanisms, and genetic redundancy of multiple copies of sRNA biosynthetic genes in the genome, warrant a detailed dissection of sRNA generating pathway(s) through the use of various sRNA biosynthetic mutants. Further studies on *in planta* interaction using sRNA biosynthetic mutants would provide better understanding of molecular mechanisms behind host-pathogen interaction which in turn useful for developing novel disease control strategies.

## Methods

### Fungal strain and growth conditions

*M. oryzae* wild type strain 70–15 was used in this study because of the availability of genome sequence and transcriptome data. Filter paper stocks stored at -20°C were inoculated onto Oatmeal agar plates (OM: oatmeal 50 g/L and agar 15 g/L) for germination and to establish starter cultures. Fungus grown on OM plates were transferred to complete medium (CM: 10 g sucrose, 6 g yeast extract, 6 g casamino acid and 1 mL trace elements/L) plates and subsequently to complete liquid medium. Liquid grown fungus was harvested under sterile conditions and washed with sterile distilled water. The resulting fungal mat was carefully divided into equal pieces for further exposure to physiological conditions in liquid such as complete medium (CM), minimal medium (MM), carbon starved medium (CS: MM lacking carbon source), nitrogen starved medium (NS: MM lacking nitrogen source) and a reactive oxygen species-rich environment generated by amendment of Paraquat (PQ) to the CM, as described in detail by Mathioni et al. [29]. For the *in planta* libraries, twenty-day-old rice (Nipponbare) seedlings were mock inoculated with 0.2% gelatin and leaves were harvested immediately (LMg0). Conidia from *M. oryzae* 70–15 grown on OM plates for 10 days (d) were harvested using 0.2% gelatin. Concentration of the conidial suspension was adjusted to 1 × 10^5^ spores/mL and 3 mL of the suspension was spray inoculated onto rice plants; leaves were harvested after 72 hours post-inoculation (hpi) and 96 hpi respectively for LMg72 and LMg96 libraries.

### Small RNA library construction and data analysis

Total RNA was isolated using Trizol reagent (Invitrogen, New York, USA) according to manufacturer’s instructions. The quality of the RNA was checked by agarose gel electrophoresis and NanoDrop ND-1000. High molecular weight RNA was removed by PEG precipitation, and low molecular weight RNA was size-fractionated on a polyacrylamide gel. Gel portions corresponding to 20–30 nt were recovered and small RNA libraries were constructed by following the protocol by Lu et al. [83] with modifications using standard Illumina sRNA adapters. The quality of the resulting libraries was checked by subjecting an aliquot of each library to TOPO® cloning (Invitrogen, USA), and approximately 96 clones from each library were sequenced by the Sanger method. The resulting sequences were checked for the presence of adapters, size of cDNAs, and their genome match. The libraries were then sequenced using Illumina’s Genome Analyzer II (GA_II_) at the Delaware Biotechnology Institute. Adapters were removed from the resulting sequences and matched to the *M. oryzae* genome (version 6 assembly) from the Broad Institute, and reads with sequencing errors were filtered out using PERL scripts. tRNA, rRNA, snRNA or snoRNAs -matched reads were removed from the total reads, and genome-matched reads were obtained using the program Bowtie, accepting only perfect matches. From the total genome matched reads, distinct genome matched reads were calculated. The abundance of each read was normalized to 5 million units of transcripts per 5 million (TP5M) to account for the difference in the sequencing depth and to facilitate accurate comparisons across libraries. To compare the different libraries, the entire genome was divided arbitrarily into 500 bp windows/clusters based on the proximity-based algorithm ([30]; described in the Results section). Clusters showing ≥ 10-fold difference between 2 or more libraries were selected for further cluster analysis. Repeat analysis was performed using programs namely, RepeatMasker [31], etandem and einverted [84].

### Targeted disruption of genes involved in sRNA biogenesis and mutant screening

Gene replacement cassettes were constructed using adaptamer-mediated PCR [85]. Typically, 1.3 kb of upstream and downstream sequence of each target gene was amplified with primers that contained adaptamer sequences. A 1.4 kb fragment containing the hygromycin B phosphotransferese gene with the *trp*C promoter from *A. nidulans* was amplified from plasmid pCB1003 using the adaptamer sequence attached to the forward HPHF and reverse HPHR primer set (Additional file 11: Table S3). Using nested primers, three individually amplified fragments of upstream and downstream sequence and hygromycin resistance gene were combined and amplified together to construct a hygromycin cassette for gene replacement of approximately 3.3 kb in length. The hygromycin cassette was transformed into 70–15 protoplasts as previously described [86]. Gene disruption mutants were identified by PCR screening using primers outside the flanking regions and gene specific primers (Additional file 11: Table S3) and further confirmed by Southern blot analysis (Additional file 10: Figure S8). For deletion of *MoDcl1* and *MoDcl2*, gene replacement cassettes were constructed as described previously with the modification of BAR gene, amplified from pCB1530, being used as the selectable marker [87] in place of HYG. Gene replacement cassettes for *MoDcl1* and *MoDcl2* were transformed into protoplasts of *MoDcl2* and *MoDcl1* knockouts, respectively.

### Quantitative reverse transcription-polymerase chain reaction (qRT-PCR)

Genomic DNA was removed from total RNA by TURBO® DNase treatment (Ambion, New York, USA). Reverse transcription reactions were performed with 500 ng of RNA in 20 μl reaction using High Capacity RNA-to-cDNA Kit (Applied Biosystems, California, USA) by following the manufacturer’s instructions. Real time PCR was done using 5 PRIME SYBR mastermix for Real-time PCR (5 PRIME, Maryland, USA) in an Eppendorf Mastercycler using 2 μl of diluted reverse transcribed templates. All samples were repeated three times. Relative expression values were calculated using the 2^-ΔΔCT^ method with GADPH (MGG_01084.6) as a housekeeping control. Primers used are listed in Additional file 12: Table S4.

### GEO accession numbers

The data obtained from small RNA deep sequencing studies were deposited in the Gene Expression Omnibus database at NCBI (http://www.ncbi.nlm.nih.gov/geo/). The accession number for GEO is GSE43277). The data can also be found at http://mpss.udel.edu/mg_sRNA.

## Competing interests

The authors declare that they have no competing interests.

## Authors’ contributions

BCM and ND designed the study and NMD and VR wrote the manuscript. VR and SAS generated the libraries. FD and JZ performed bioinformatic analyses. MR generated one mutant and SMM provided guidance on microarray comparisons. Mutant generation and molecular analyses were performed by VR SAS, VR, NMD, BCM, SMM and MR edited the manuscript. All authors read and approved the final manuscript.

## Supplementary Material

Additional file 1: Figure S1 Informatics pipeline used for the analysis sRNA libraries of *M. oryzae* different libraries. We used a pipeline with different filtering steps to analyze the raw reads obtained from Illumina. The filtering steps (left column) are summarized here and fully described in the main text. Results of each filtering step to the *M.* oryzae mycelial complete media (CM) library are shown in the right column, as an example. The colors of the numbers indicate either total (black) or distinct (red) sequences. Click here for file

Additional file 2: Figure S2 sRNAs associate with the retrotransposon class, LTR/Gypsy. Size distribution of representative loci from the major retrotransposon classes LTR/Gypsy in mycelial libraries (A); One representative locus of GYMAG1_I-int LTR/Gypsy on chromosome 6 under wild type (complete media) conditions (B); abundance of 16 different LTR/Gypsy retrotransposons on sense (open box) and antisense (filled box) strands of all mycelial libraries (C); abundance of sRNAs from LTR/Gypsy classes under different stresses in the mycelial libraries (D). (CM = complete media; CS = carbon starved; MM = minimal media; NS = nitrogen starved; PQ = paraquat). Click here for file

Additional file 3: Figure S3 sRNAs associate with the retrotransposon class, LINE/Tad1. Size distribution of representative LINE/Tad1 from different chromosomes in mycelial libraries (A); abundance of sRNAs from six LINE/Tad1 loci on sense (open box) and antisense (closed box) strands of all mycelial libraries (B); and abundance of sRNAs from LINE/Tad1 classes under different stresses from the mycelial libraries (C). (CM = complete media; CS = carbon starved; MM = minimal media; NS = nitrogen starved; PQ = paraquat). Click here for file

Additional file 4: Figure S4 Characterization of sRNAs associated with 5S rRNA and tRNA. sRNA size distribution (A and B) and abundance under different stress conditions (C and D) for 5S rRNA (left panels) and tRNA (right panels). Click here for file

Additional file 5: Figure S5 sRNAs associated with different genomic regions. Fraction of small RNA reads that associate with different genomic loci in mycelial libraries (A) and in *in planta* libraries (B). Proportion of individual repeat classes to total repeats in mycelial libraries (C) and in *in planta* libraries (D). (CM = complete media; CS = carbon starved; MM = minimal media; NS = nitrogen starved; PQ = paraquat; LMg0 = mock inoculated rice; LMg72 = 72 hpi; LMg96 = 96 hpi). Click here for file

Additional file 6: Figure S6 Screenshot representing sRNAs associated with MGG_01439, the putative inorganic phosphate transporter, and its neighboring intergenic regions under CS (carbon starved) and NS (nitrogen starved) conditions. Click here for file

Additional file 7: Table S1 List of genes showing negative correlation between sRNA and microarray data under PQ condition. Click here for file

Additional file 8: Table S2 List of genes showing negative correlation between sRNA and microarray data under NS condition. Click here for file

Additional file 9: Figure S7 Venn diagram depicting the clustering of sRNAs from LMg0, LMg72 and LMg96 libraries. The center gray area likely represents small RNAs from rice that were also found in the inoculated samples. (LMg0 = mock inoculated rice; LMg72 = 72 hpi; LMg96 = 96 hpi). Click here for file

Additional file 10: Figure S8 Generation of knock-out mutants in sRNA pathways. Schematic representation of the chromosomal locus of wild type (WT; top panel) and the deletion mutant *MoDcl1* (middle panel), as well as *MoDcl2* in the *MoDcl1* mutant background (lower panel) (A); Confirmation of the deletion of *MoDcl1* and the double mutant, using flanking, gene-specific and hygromycin/BAR-specific primers. KO = knockout; ECT = ectopic (B); Southern analysis of ∆*modcl1*, ∆*modcl2* and ∆*modcl2/modcl1* mutants. 1kb ladder (1), 70–15 (2), ∆*modcl2* KO (3), and Ectopic (5) mutants genomic DNA digested with HindIII, and 70–15 (7), ∆*modcl1* KO (8) and Ectopic (11) mutants genomic DNA digested with EcoRI and HindIII (C); ∆*modcl2/modcl1* KO1 (2), KO2 (3), Ectopic (4) mutants genomic DNA digested with EcoRI (D). All knock-out mutants were performed in the same way, with the same confirmations. Click here for file

Additional file 11: Table S3 List of primers used to generate targeted deletions. Click here for file

Additional file 12: Table S4 Primers used for qRT-PCR to study the gene expression in different mutants. Click here for file
