# Supplementary material for: Physiological stressors and invasive plant infections alter the small RNA transcriptome of the rice blast fungus, Magnaporthe oryzae
Source: BMC Genomics. 2013 May 12;14:326. doi: 10.1186/1471-2164-14-326 (PMC3658920; doi:10.1186/1471-2164-14-326)
Supplement: Additional file 1: Figure S1 — Informatics pipeline used for the analysis sRNA libraries of M. oryzae different libraries. We used a pipeline with different filtering steps to analyze the raw reads obtained from Illumina. The filtering steps (left column) are summarized here and fully described in the main text. Results of each filtering step to the M. oryzae mycelial complete media (CM) library are shown in the right column, as an example. The colors of the numbers indicate either total (black) or distinct (red) sequences. [file 1471-2164-14-326-S1.pptx]

## Slide 1
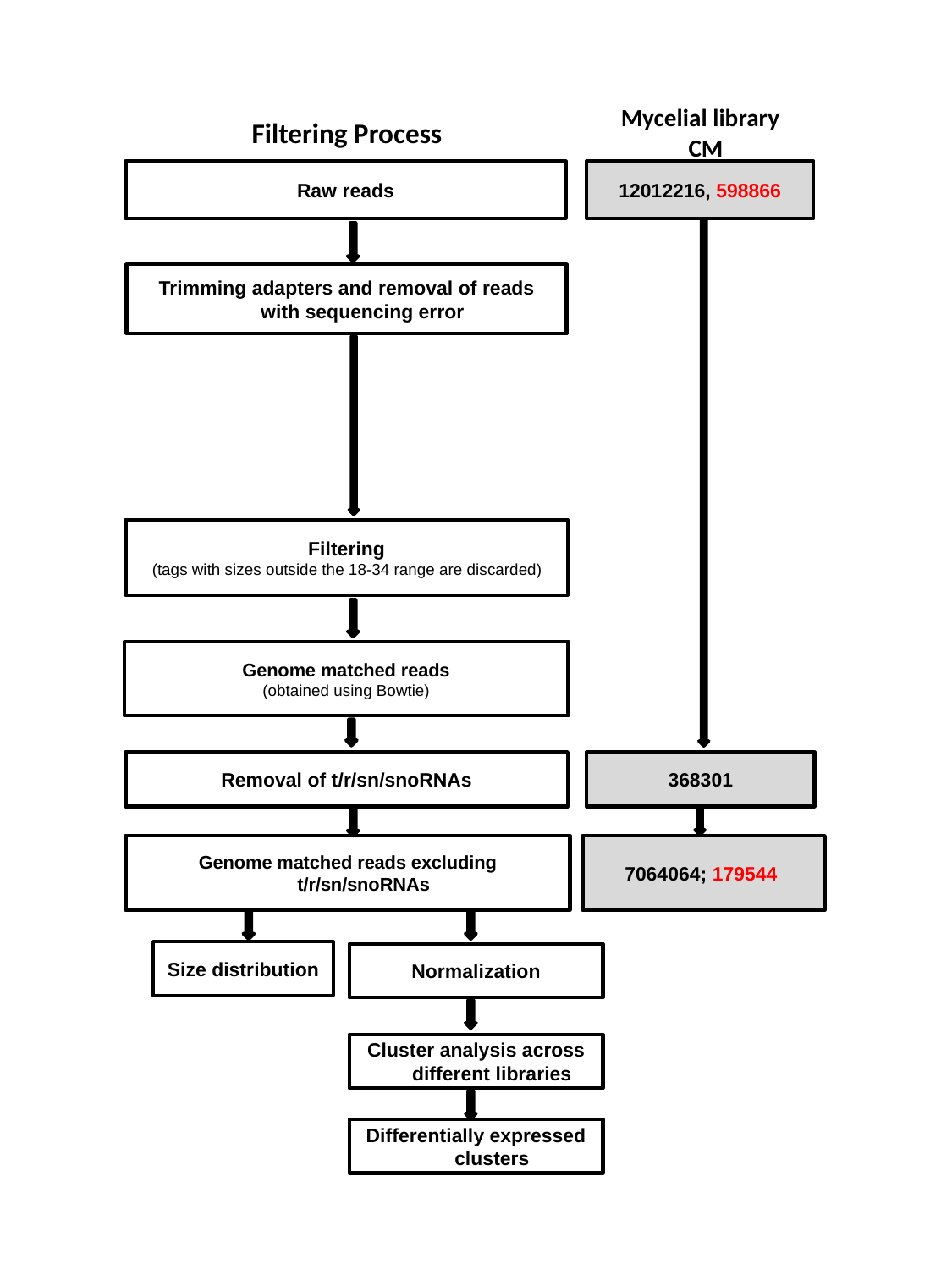

Mycelial library
CM
Filtering Process
Raw reads
12012216, 598866
Trimming adapters and removal of reads with sequencing error
Filtering
(tags with sizes outside the 18-34 range are discarded)
Genome matched reads
(obtained using Bowtie)
Removal of t/r/sn/snoRNAs
368301
Genome matched reads excluding t/r/sn/snoRNAs
7064064; 179544
Size distribution
Normalization
Cluster analysis across different libraries
Differentially expressed clusters
