# Supplementary material for: Physiological stressors and invasive plant infections alter the small RNA transcriptome of the rice blast fungus, Magnaporthe oryzae
Source: BMC Genomics. 2013 May 12;14:326. doi: 10.1186/1471-2164-14-326 (PMC3658920; doi:10.1186/1471-2164-14-326)
Supplement: Additional file 2: Figure S2 — sRNAs associate with the retrotransposon class, LTR/Gypsy. Size distribution of representative loci from the major retrotransposon classes LTR/Gypsy in mycelial libraries (A); One representative locus of GYMAG1_I-int LTR/Gypsy on chromosome 6 under wild type (complete media) conditions (B); abundance of 16 different LTR/Gypsy retrotransposons on sense (open box) and antisense (filled box) strands of all mycelial libraries (C); abundance of sRNAs from LTR/Gypsy classes under different stresses in the mycelial libraries (D). (CM = complete media; CS = carbon starved; MM = minimal media; NS = nitrogen starved; PQ = paraquat). [file 1471-2164-14-326-S2.pptx]

## Slide 1
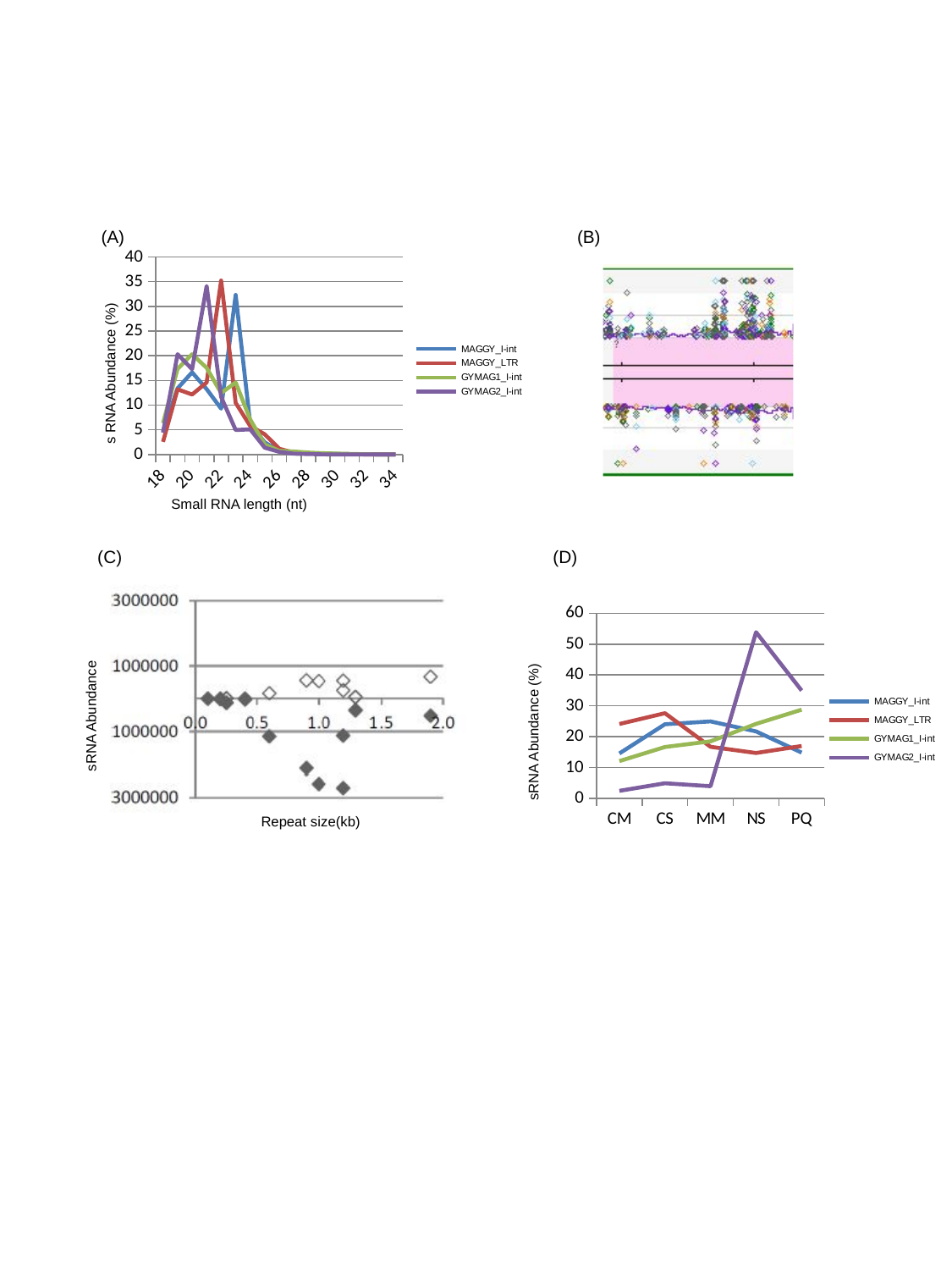

(A)
(B)
### Chart
| Category | MAGGY_I-int | MAGGY_LTR | GYMAG1_I-int | GYMAG2_I-int |
|---|---|---|---|---|
| 18 | 4.734602495306742 | 2.5563987480068566 | 6.330759437482637 | 4.415887114554789 |
| 19 | 13.393803611908355 | 13.202031536053859 | 17.34331022843119 | 20.321899902359128 |
| 20 | 16.629880477965056 | 12.150091537235006 | 20.30394084104445 | 17.28558379791489 |
| 21 | 13.24308947367285 | 14.575090060827998 | 17.552571375611564 | 34.11761000346456 |
| 22 | 9.284304774881141 | 35.24109726569404 | 12.445184798567874 | 11.650760653878862 |
| 23 | 32.33205974040121 | 10.458867300537412 | 14.581209422117293 | 4.9607861664934285 |
| 24 | 6.2208814469295755 | 5.772013228606863 | 7.080002146268178 | 5.083624681092338 |
| 25 | 2.4312116733607967 | 4.0859564164648905 | 2.0233455442984876 | 1.3701218936029478 |
| 26 | 0.7635095777942503 | 1.1560266934388472 | 0.7468037676762257 | 0.5323002299285017 |
| 27 | 0.29801274577589765 | 0.3631961259079911 | 0.60315013633681 | 0.15433556962424014 |
| 28 | 0.22551733751074346 | 0.2605858383039038 | 0.39096225982527566 | 0.08189234306592362 |
| 29 | 0.21804009463789462 | 0.0752967578101934 | 0.2541376635919749 | 0.025197644020284148 |
| 30 | 0.11514338613251461 | 0.04355400696864113 | 0.17340871287321896 | 0.0 |
| 31 | 0.0668028571068118 | 0.0339573613653812 | 0.1034111030355061 | 0.0 |
| 32 | 0.025970341500759688 | 0.01993149471446297 | 0.04829103396469391 | 0.0 |
| 33 | 0.012615924188757621 | 0.0029528140317722808 | 0.009755764437311942 | 0.0 |
| 34 | 0.004554040926673479 | 0.0029528140317722808 | 0.009755764437311942 | 0.0 |
s RNA Abundance (%)
Small RNA length (nt)
(C)
(D)
### Chart
| Category | MAGGY_I-int | MAGGY_LTR | GYMAG1_I-int | GYMAG2_I-int |
|---|---|---|---|---|
| CM | 14.537452524892602 | 24.09570070276967 | 12.057880950406572 | 2.4095247094396672 |
| CS | 24.01890419313318 | 27.601429161991376 | 16.62723711873253 | 4.866295001417369 |
| MM | 24.94429761765195 | 16.685613890037107 | 18.456930738950376 | 3.915083939651635 |
| NS | 21.690558457730965 | 14.672532923876474 | 24.127956606359795 | 53.856814387854726 |
| PQ | 14.808787206591306 | 16.944723321325124 | 28.729994585550727 | 34.95228196163631 |sRNA Abundance (%)
sRNA Abundance
Repeat size(kb)
