# Supplementary material for: Physiological stressors and invasive plant infections alter the small RNA transcriptome of the rice blast fungus, Magnaporthe oryzae
Source: BMC Genomics. 2013 May 12;14:326. doi: 10.1186/1471-2164-14-326 (PMC3658920; doi:10.1186/1471-2164-14-326)
Supplement: Additional file 3: Figure S3 — sRNAs associate with the retrotransposon class, LINE/Tad1. Size distribution of representative LINE/Tad1 from different chromosomes in mycelial libraries (A); abundance of sRNAs from six LINE/Tad1 loci on sense (open box) and antisense (closed box) strands of all mycelial libraries (B); and abundance of sRNAs from LINE/Tad1 classes under different stresses from the mycelial libraries (C). (CM = complete media; CS = carbon starved; MM = minimal media; NS = nitrogen starved; PQ = paraquat). [file 1471-2164-14-326-S3.pptx]

## Slide 1
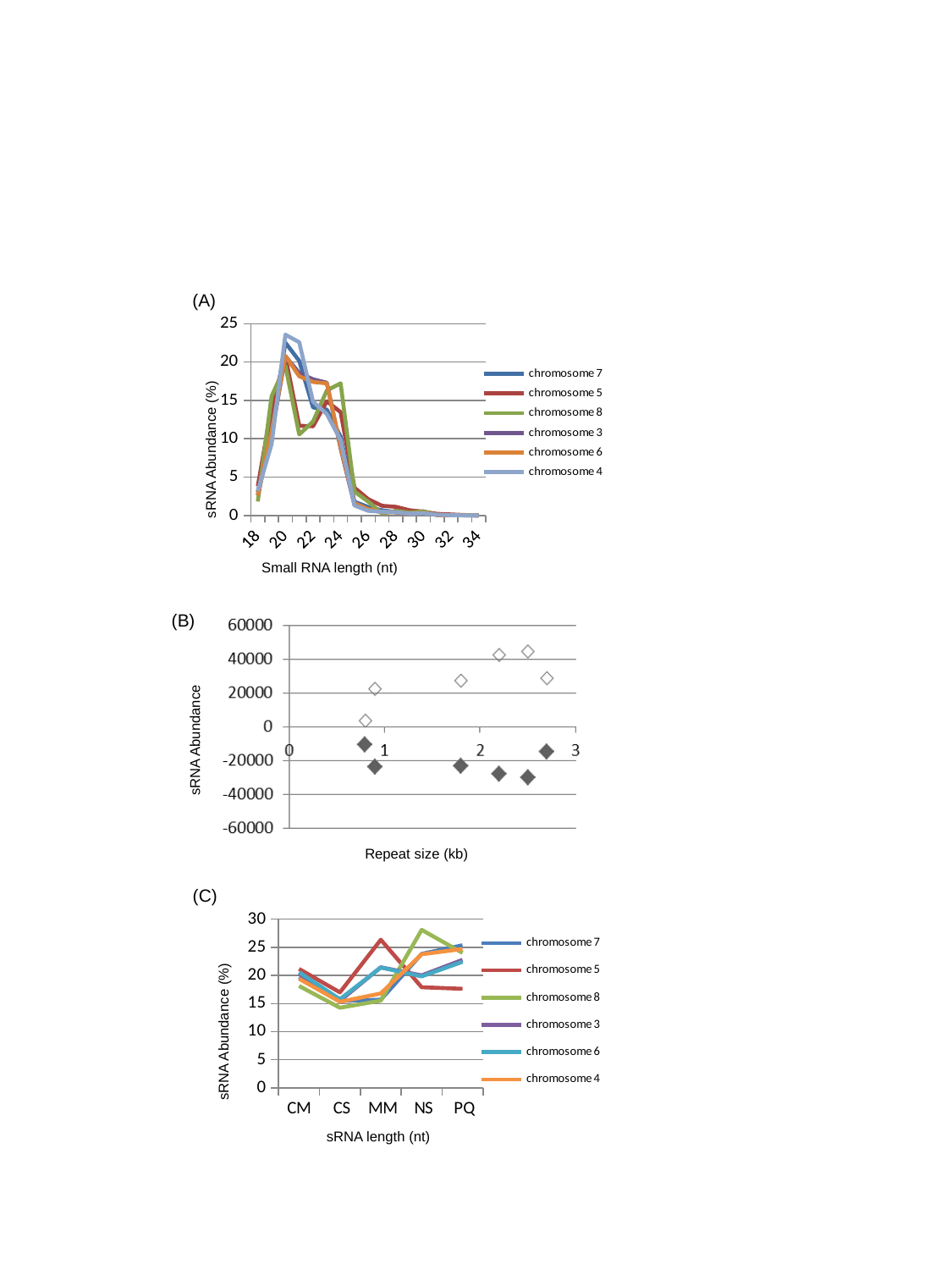

(A)
### Chart
| Category | | | | | | |
|---|---|---|---|---|---|---|
| 18 | 3.044399136958375 | 3.8221546821394847 | 1.821608040201005 | 2.654414065834021 | 2.6156779547224542 | 3.2036563704891043 |
| 19 | 11.409568677131377 | 14.112039787248754 | 15.549972082635398 | 10.60627613872372 | 10.924617987485565 | 9.296885153576225 |
| 20 | 22.52419881628694 | 20.738642904837487 | 19.500279173646007 | 20.751657230646675 | 20.81666084808121 | 23.575791708183512 |
| 21 | 20.14885488628046 | 11.687504317192802 | 10.559743160245672 | 18.57378588295542 | 18.14055912130409 | 22.58155352423862 |
| 22 | 14.091727864763753 | 11.63224424949923 | 12.241764377442768 | 17.75157187971208 | 17.406074603217228 | 14.829095004982022 |
| 23 | 13.796789326787971 | 14.890285740600032 | 16.289782244556047 | 17.302056957523686 | 17.226146037543206 | 13.221851579084168 |
| 24 | 10.273362497278251 | 13.471944003131403 | 17.23199329983253 | 8.755583373637972 | 8.917206004780192 | 9.818914352553819 |
| 25 | 1.785466854054907 | 3.6149294282885487 | 3.112786152987159 | 1.37557256251956 | 1.5992158337137834 | 1.303989949313348 |
| 26 | 1.0946376610780129 | 2.118302594920679 | 1.8006700167504188 | 0.7013001792369611 | 0.825791551413918 | 0.5978425681237274 |
| 27 | 0.6670757536768375 | 1.2525615343878795 | 0.25125628140703515 | 0.4680076246834903 | 0.4310229073233627 | 0.48087337001256436 |
| 28 | 0.42558245412617035 | 1.1190163707950542 | 0.6002233389168062 | 0.419642095080942 | 0.4014823965410748 | 0.4288870597409349 |
| 29 | 0.2771234585007621 | 0.667725817964131 | 0.36990508096035807 | 0.22475746109419933 | 0.22826758331766794 | 0.23177229996101026 |
| 30 | 0.30087689780082943 | 0.4950381064216804 | 0.551367950865437 | 0.3072633645338424 | 0.3464296264468138 | 0.2945890915392289 |
| 31 | 0.09897266375027258 | 0.20031774538923824 | 0.034896705750977156 | 0.06828074767418701 | 0.07250852646561216 | 0.09097604297535018 |
| 32 | 0.0376096122251036 | 0.12894015795169386 | 0.055834729201563404 | 0.019915218071637885 | 0.02685500980207861 | 0.034657540181085654 |
| 33 | 0.019794532750054435 | 0.048352559231885084 | 0.013958682300390839 | 0.011380124612364574 | 0.013427504901039305 | 0.008664385045271412 |
| 34 | 0.003958906550010889 | 0.0 | 0.013958682300390839 | 0.008535093459273401 | 0.008056502940623602 | 0.0 |sRNA Abundance (%)
Small RNA length (nt)
(B)
sRNA Abundance
Repeat size (kb)
(C)
### Chart
| Category | | | | | | |
|---|---|---|---|---|---|---|
| CM | 19.679724460104133 | 21.14848840689833 | 18.12534896705753 | 20.30071979288173 | 20.572280258882287 | 19.41038859766928 |
| CS | 15.437756091767454 | 16.987865810135617 | 14.24483528754885 | 15.482659535121961 | 15.773290007250848 | 15.309968374994583 |
| MM | 15.683208297868129 | 26.347539775736173 | 15.515075376884422 | 21.44299980084776 | 21.407471063726927 | 16.795910410258635 |
| NS | 23.83459688434054 | 17.895051921438654 | 28.105806811836963 | 20.001991521807195 | 19.8230254854043 | 23.77074037170204 |
| PQ | 25.364714265919726 | 17.621054085791254 | 24.00893355667223 | 22.77162934934129 | 22.42393318473557 | 24.71299224537531 |sRNA Abundance (%)
sRNA length (nt)
