# Supplementary material for: Physiological stressors and invasive plant infections alter the small RNA transcriptome of the rice blast fungus, Magnaporthe oryzae
Source: BMC Genomics. 2013 May 12;14:326. doi: 10.1186/1471-2164-14-326 (PMC3658920; doi:10.1186/1471-2164-14-326)
Supplement: Additional file 4: Figure S4 — Characterization of sRNAs associated with 5S rRNA and tRNA. sRNA size distribution (A and B) and abundance under different stress conditions (C and D) for 5S rRNA (left panels) and tRNA (right panels). [file 1471-2164-14-326-S4.pptx]

## Slide 1
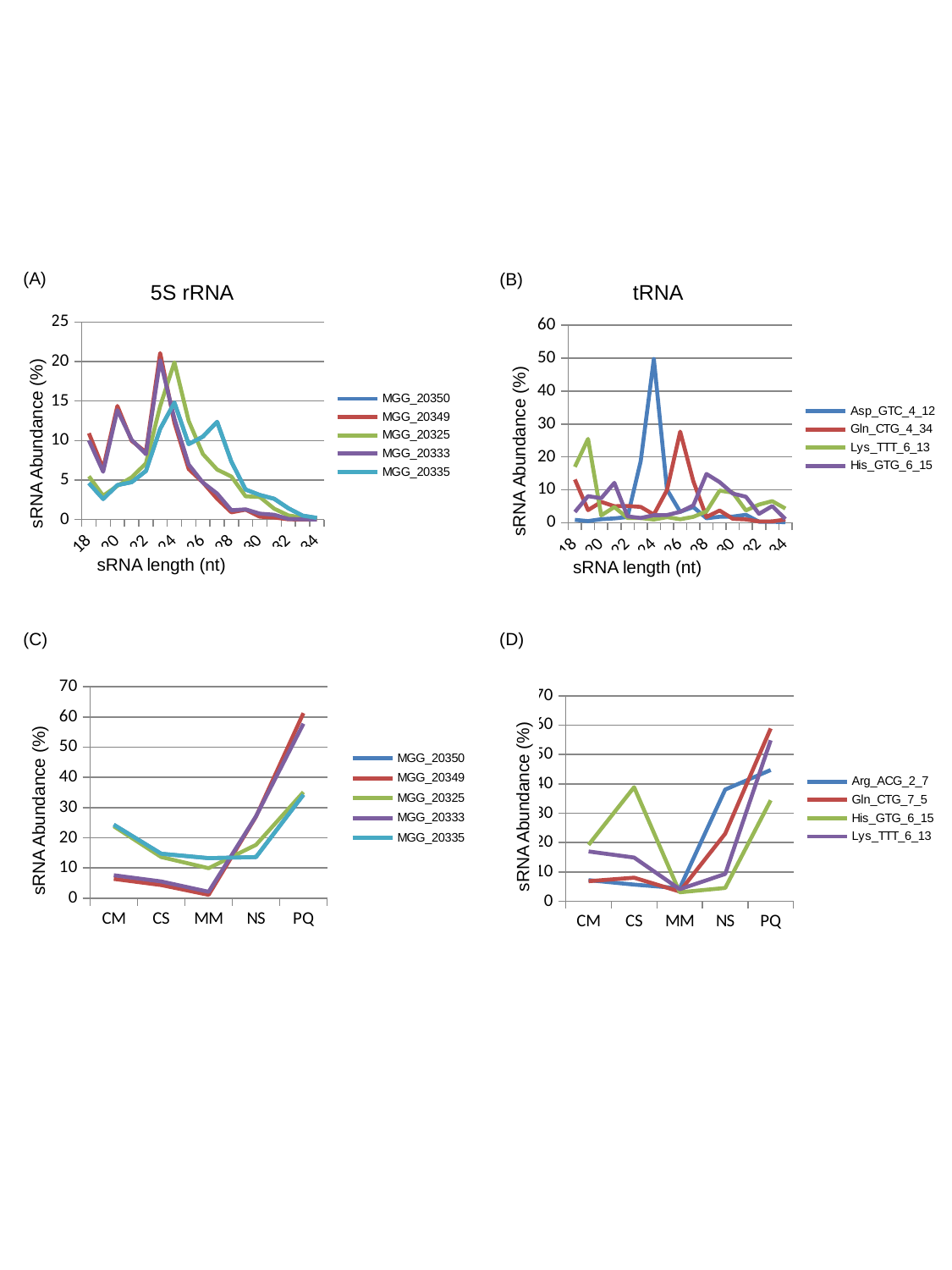

(A)
(B)
5S rRNA
tRNA
### Chart
| Category | MGG_20350 | MGG_20349 | MGG_20325 | MGG_20333 | MGG_20335 |
|---|---|---|---|---|---|
| 18 | 4.603165344532587 | 10.9227321445971 | 5.466597452381903 | 10.011436614762106 | 4.603165344532587 |
| 19 | 2.591771659667135 | 6.440611906849849 | 3.0094903432762314 | 6.043811031934547 | 2.591771659667135 |
| 20 | 4.3542930471301915 | 14.372015979733026 | 4.258009556895442 | 13.847101258027624 | 4.3542930471301915 |
| 21 | 4.721768548763416 | 9.958101919516713 | 5.3388172343568945 | 10.090613178499165 | 4.721768548763416 |
| 22 | 6.136257582827811 | 8.516028451719759 | 7.105112539764912 | 8.269552212545152 | 6.136257582827811 |
| 23 | 11.479234717685552 | 21.065965117412194 | 14.353977824808052 | 20.163631565056743 | 11.479234717685552 |
| 24 | 14.799152278736972 | 12.257624476273994 | 19.89245164982896 | 12.765021553620128 | 14.799152278736972 |
| 25 | 9.540752838699657 | 6.391893208613467 | 12.491847355881267 | 6.967537608867744 | 9.540752838699657 |
| 26 | 10.486662000311092 | 4.686738770340057 | 8.280424336807377 | 4.671417260490895 | 10.486662000311092 |
| 27 | 12.362925804946395 | 2.621065965117414 | 6.321127660424071 | 3.281428697105657 | 12.362925804946395 |
| 28 | 7.310623736195367 | 0.906167787196723 | 5.431990310000153 | 1.1700536641154282 | 7.310623736195367 |
| 29 | 3.787525276092726 | 1.2374549352041257 | 2.9362829266993953 | 1.2844198117357317 | 3.787525276092726 |
| 30 | 3.098265671177477 | 0.35077462730196035 | 2.843109851056185 | 0.7389812615465862 | 3.098265671177477 |
| 31 | 2.6219085394307027 | 0.25333723082919224 | 1.3709752558931958 | 0.5982229260138999 | 2.6219085394307027 |
| 32 | 1.4212941359464863 | 0.01948747929455343 | 0.5137829599755085 | 0.07037916776634116 | 1.4212941359464863 |
| 33 | 0.48705086327578473 | 0.0 | 0.24624312848567212 | 0.02639218791237794 | 0.48705086327578473 |
| 34 | 0.19734795458080653 | 0.0 | 0.13975961346484048 | 0.0 | 0.19734795458080653 |
### Chart
| Category | Asp_GTC_4_12 | Gln_CTG_4_34 | Lys_TTT_6_13 | His_GTG_6_15 |
|---|---|---|---|---|
| 18 | 0.8633093525179888 | 13.115045822582086 | 16.959459459459495 | 3.2651941281382912 |
| 19 | 0.4759269507470962 | 3.808167986475665 | 25.45608108108108 | 8.0532308958705 |
| 20 | 1.0514665190924128 | 6.308390426194501 | 2.212837837837838 | 7.4358622581972815 |
| 21 | 1.3060320973990038 | 5.0004448794376675 | 4.74662162162166 | 12.100425298394844 |
| 22 | 1.7266187050359698 | 5.062728000711807 | 1.4358108108108099 | 1.920702428316649 |
| 23 | 18.69396790260103 | 4.786902749354931 | 1.402027027027027 | 1.4130882151186657 |
| 24 | 49.7399003873824 | 2.4646320847050447 | 0.9459459459459455 | 2.277404307861161 |
| 25 | 10.027670171555048 | 9.911913871340861 | 1.6722972972972974 | 2.3185622170393745 |
| 26 | 3.298284449363586 | 27.66260343446912 | 1.0472972972972892 | 3.2926327342571 |
| 27 | 4.748201438848922 | 12.581190497375212 | 1.7736486486486478 | 5.26821237481136 |
| 28 | 1.3392363032650798 | 1.7172346294154226 | 3.429054054054054 | 14.803128001097543 |
| 29 | 1.793027116768124 | 3.7013969214342914 | 9.746621621621614 | 12.319934147345375 |
| 30 | 1.848367459878258 | 1.18337930420856 | 9.121621621621614 | 8.848950473315954 |
| 31 | 2.3907028223574986 | 0.9876323516327076 | 3.7162162162162162 | 7.8748799560982246 |
| 32 | 0.2877697841726636 | 0.37369872764481077 | 5.5067567567567455 | 2.6752640965838927 |
| 33 | 0.27670171555063644 | 0.39149390515170546 | 6.53716216216219 | 5.048703525860891 |
| 34 | 0.13281682346430548 | 0.9431444078654686 | 4.2905405405405395 | 1.0838249416929564 |sRNA Abundance (%)
sRNA Abundance (%)
sRNA length (nt)
sRNA length (nt)
(C)
(D)
### Chart
| Category | MGG_20350 | MGG_20349 | MGG_20325 | MGG_20333 | MGG_20335 |
|---|---|---|---|---|---|
| CM | 24.28449214496799 | 6.3431745103770645 | 23.784424123840328 | 7.548829843392579 | 24.28449214496799 |
| CS | 14.665966713330224 | 4.316476663743545 | 13.575317121218168 | 5.4988562379025145 | 14.665966713330224 |
| MM | 13.219396484678798 | 1.0620676215531561 | 9.883001237870863 | 2.076368115431978 | 13.219396484678798 |
| NS | 13.580066884429987 | 26.99015882295625 | 17.640325307138387 | 27.107161710364245 | 13.580066884429987 |
| PQ | 34.250077772592924 | 61.28812238137019 | 35.11693220993225 | 57.76878409290868 | 34.250077772592924 |
### Chart
| Category | Arg_ACG_2_7 | Gln_CTG_7_5 | His_GTG_6_15 | Lys_TTT_6_13 |
|---|---|---|---|---|
| CM | 7.171560627048677 | 6.814564265817714 | 19.193304980107012 | 16.942567567567423 |
| CS | 5.6574273449178945 | 8.008356545961004 | 38.85306626423338 | 14.881756756756756 |
| MM | 4.421708063034051 | 3.2928770393951377 | 3.045685279187817 | 4.087837837837812 |
| NS | 38.04808470368775 | 23.020294468762437 | 4.5273700096035085 | 9.273648648648653 |
| PQ | 44.701219261311394 | 58.86390768006344 | 34.380573466867844 | 54.814189189189094 |sRNA Abundance (%)
sRNA Abundance (%)
