# Supplementary material for: Physiological stressors and invasive plant infections alter the small RNA transcriptome of the rice blast fungus, Magnaporthe oryzae
Source: BMC Genomics. 2013 May 12;14:326. doi: 10.1186/1471-2164-14-326 (PMC3658920; doi:10.1186/1471-2164-14-326)
Supplement: Additional file 5: Figure S5 — sRNAs associated with different genomic regions. Fraction of small RNA reads that associate with different genomic loci in mycelial libraries (A) and in in planta libraries (B). Proportion of individual repeat classes to total repeats in mycelial libraries (C) and in in planta libraries (D). (CM = complete media; CS = carbon starved; MM = minimal media; NS = nitrogen starved; PQ = paraquat; LMg0 = mock inoculated rice; LMg72 = 72 hpi; LMg96 = 96 hpi). [file 1471-2164-14-326-S5.pptx]

## Slide 1
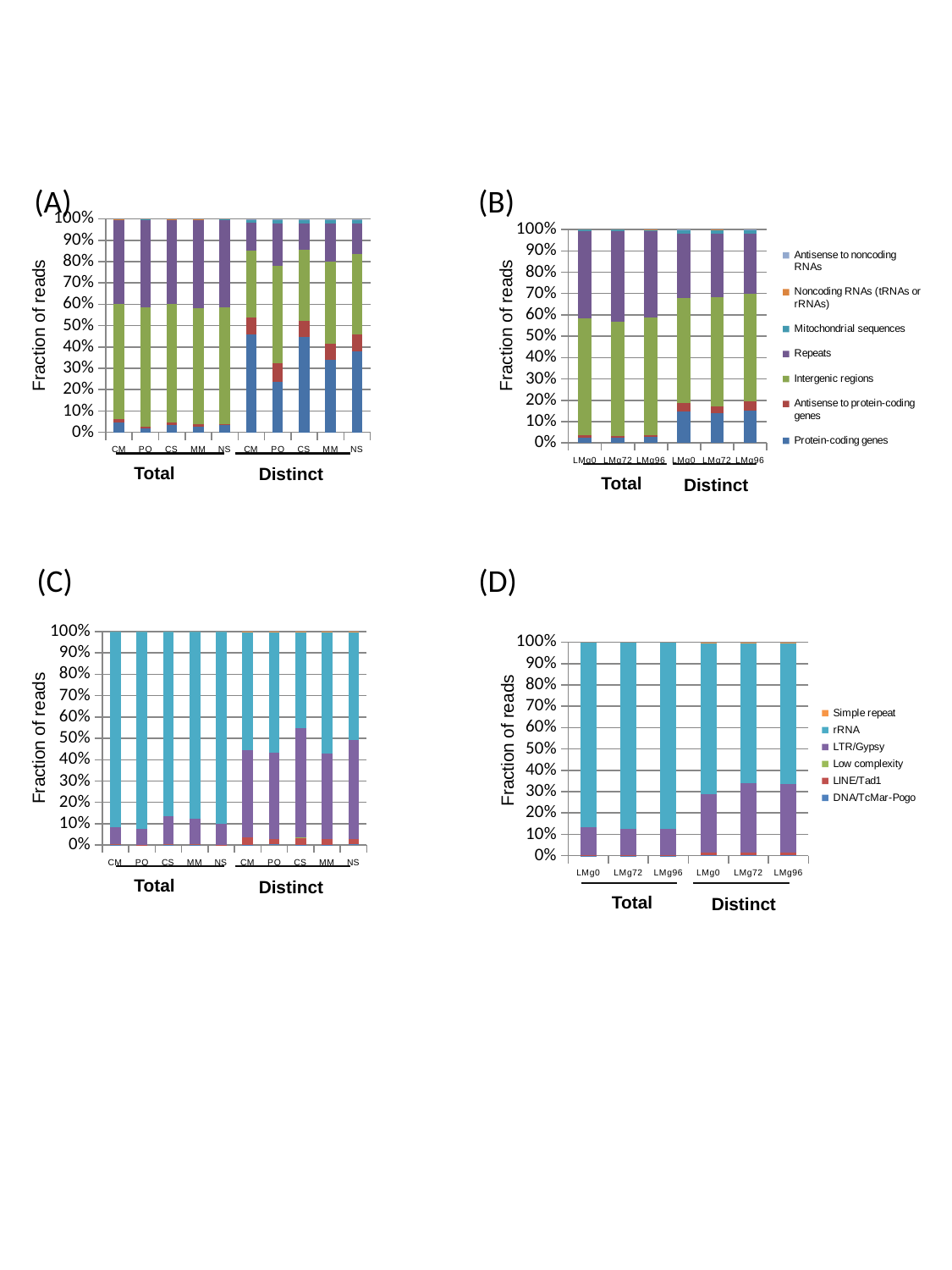

(A)
(B)
### Chart
| Category | Protein-coding genes | Antisense to protein-coding genes | Intergenic regions | Repeats | Mitochondrial sequences | Noncoding RNAs (tRNAs or rRNAs) | Antisense to noncoding RNAs |
|---|---|---|---|---|---|---|---|
| CM | 398626.0 | 117594.0 | 4577671.0 | 3293229.0 | 53894.0 | 3069.0 | 294.0 |
| PQ | 170622.0 | 53300.0 | 4874842.0 | 3572048.0 | 41742.0 | 829.0 | 350.0 |
| CS | 307976.0 | 96637.0 | 4767466.0 | 3373153.0 | 46566.0 | 2129.0 | 278.0 |
| MM | 242277.0 | 83657.0 | 4734985.0 | 3575400.0 | 54120.0 | 2236.0 | 354.0 |
| NS | 283152.0 | 65186.0 | 4792313.0 | 3569899.0 | 45513.0 | 1558.0 | 325.0 |
| CM | 97998.0 | 17261.0 | 67163.0 | 27318.0 | 2846.0 | 1117.0 | 126.0 |
| PQ | 36724.0 | 13720.0 | 71458.0 | 30727.0 | 2916.0 | 366.0 | 147.0 |
| CS | 177276.0 | 28742.0 | 132449.0 | 49699.0 | 5691.0 | 1906.0 | 261.0 |
| MM | 36531.0 | 7710.0 | 41311.0 | 19324.0 | 1650.0 | 469.0 | 77.0 |
| NS | 98104.0 | 20112.0 | 97174.0 | 37391.0 | 4180.0 | 1030.0 | 221.0 |
### Chart
| Category | Protein-coding genes | Antisense to protein-coding genes | Intergenic regions | Repeats | Mitochondrial sequences | Noncoding RNAs (tRNAs or rRNAs) | Antisense to noncoding RNAs |
|---|---|---|---|---|---|---|---|
| LMg0 | 226350.0 | 74344.0 | 4763989.0 | 3546606.0 | 54440.0 | 1605.0 | 206.0 |
| LMg72 | 221346.0 | 55355.0 | 4770350.0 | 3792791.0 | 66071.0 | 1803.0 | 203.0 |
| LMg96 | 233872.0 | 89154.0 | 4745049.0 | 3515115.0 | 56345.0 | 2068.0 | 369.0 |
| LMg0 | 3089.0 | 771.0 | 10263.0 | 6207.0 | 376.0 | 34.0 | 6.0 |
| LMg72 | 4428.0 | 1016.0 | 16150.0 | 9430.0 | 526.0 | 44.0 | 6.0 |
| LMg96 | 4409.0 | 1207.0 | 14735.0 | 8214.0 | 482.0 | 43.0 | 10.0 |Fraction of reads
Fraction of reads
Total
Distinct
Total
Distinct
(C)
(D)
### Chart
| Category | DNA/TcMar-Pogo | LINE/Tad1 | Low complexity | LTR/Gypsy | rRNA | Simple repeat |
|---|---|---|---|---|---|---|
| CM | 297.0 | 12004.0 | 5.0 | 268986.0 | 3011834.0 | 300.0 |
| PQ | 1753.0 | 3374.0 | 0.0 | 258989.0 | 3308835.0 | 94.0 |
| CS | 867.0 | 8016.0 | 5.0 | 446957.0 | 2917766.0 | 241.0 |
| MM | 835.0 | 10645.0 | 4.0 | 424605.0 | 3139922.0 | 114.0 |
| NS | 878.0 | 4029.0 | 2.0 | 349310.0 | 3215905.0 | 344.0 |
| CM | 48.0 | 947.0 | 2.0 | 11143.0 | 15097.0 | 93.0 |
| PQ | 122.0 | 698.0 | 0.0 | 12471.0 | 17418.0 | 29.0 |
| CS | 112.0 | 1688.0 | 5.0 | 25340.0 | 22372.0 | 199.0 |
| MM | 25.0 | 541.0 | 1.0 | 7683.0 | 11052.0 | 28.0 |
| NS | 115.0 | 1004.0 | 2.0 | 17293.0 | 18876.0 | 117.0 |
### Chart
| Category | DNA/TcMar-Pogo | LINE/Tad1 | Low complexity | LTR/Gypsy | rRNA | Simple repeat |
|---|---|---|---|---|---|---|
| LMg0 | 645.0 | 11198.0 | 0.0 | 468017.0 | 3067165.0 | 226.0 |
| LMg72 | 1118.0 | 9755.0 | 0.0 | 472091.0 | 3310278.0 | 387.0 |
| LMg96 | 960.0 | 9512.0 | 0.0 | 437425.0 | 3067783.0 | 182.0 |
| LMg0 | 4.0 | 90.0 | 0.0 | 1702.0 | 4409.0 | 6.0 |
| LMg72 | 11.0 | 127.0 | 0.0 | 3078.0 | 6205.0 | 11.0 |
| LMg96 | 7.0 | 118.0 | 0.0 | 2646.0 | 5437.0 | 9.0 |Fraction of reads
Fraction of reads
Total
Distinct
Total
Distinct
