# Supplementary figures and images for: Physiological stressors and invasive plant infections alter the small RNA transcriptome of the rice blast fungus, Magnaporthe oryzae
Source: BMC Genomics. 2013 May 12;14:326. doi: 10.1186/1471-2164-14-326 (PMC3658920; doi:10.1186/1471-2164-14-326)

## Slide 1
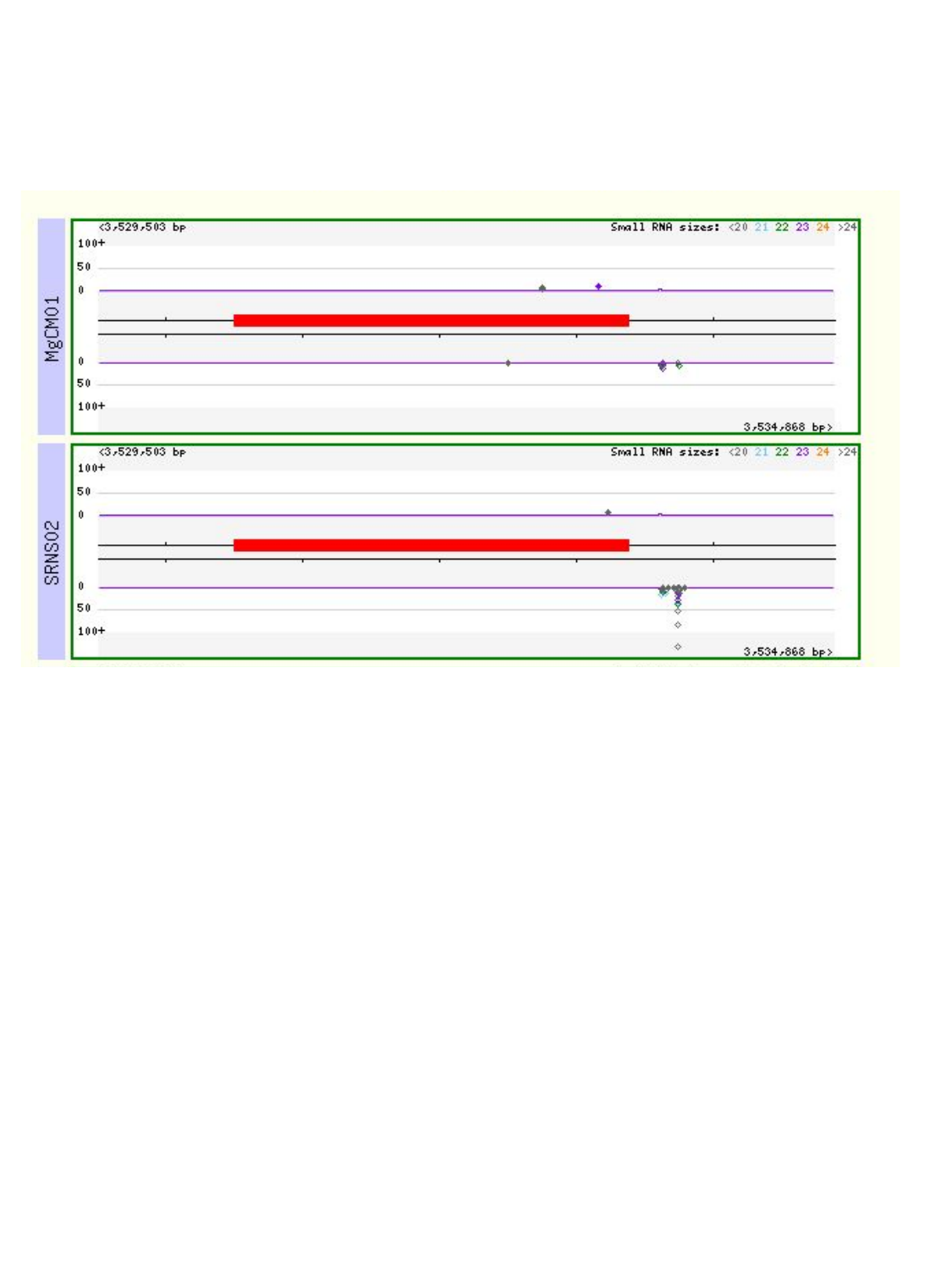

Supplement: Additional file 6: Figure S6 — Screenshot representing sRNAs associated with MGG_01439, the putative inorganic phosphate transporter, and its neighboring intergenic regions under CS (carbon starved) and NS (nitrogen starved) conditions. [file 1471-2164-14-326-S6.pptx]

## Slide 1
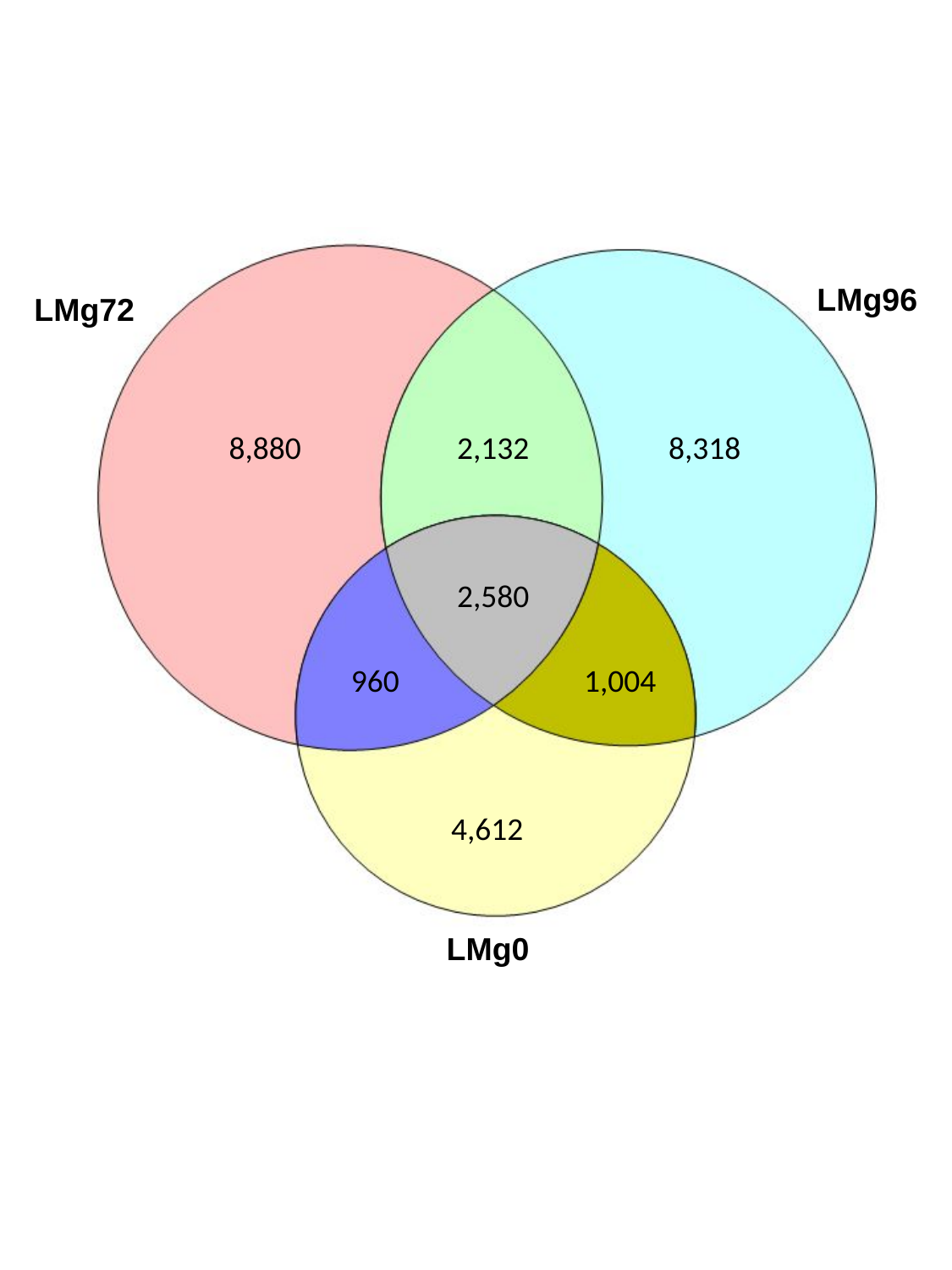

LMg96
LMg72
8,880
2,132
8,318
2,580
960
1,004
4,612
LMg0

Supplement: Additional file 9: Figure S7 — Venn diagram depicting the clustering of sRNAs from LMg0, LMg72 and LMg96 libraries. The center gray area likely represents small RNAs from rice that were also found in the inoculated samples. (LMg0 = mock inoculated rice; LMg72 = 72 hpi; LMg96 = 96 hpi). [file 1471-2164-14-326-S9.pptx]
