# Supplementary material for: Physiological stressors and invasive plant infections alter the small RNA transcriptome of the rice blast fungus, Magnaporthe oryzae
Source: BMC Genomics. 2013 May 12;14:326. doi: 10.1186/1471-2164-14-326 (PMC3658920; doi:10.1186/1471-2164-14-326)
Supplement: Additional file 10: Figure S8 — Generation of knock-out mutants in sRNA pathways. Schematic representation of the chromosomal locus of wild type (WT; top panel) and the deletion mutant MoDcl1 (middle panel), as well as MoDcl2 in the MoDcl1 mutant background (lower panel) (A); Confirmation of the deletion of MoDcl1 and the double mutant, using flanking, gene-specific and hygromycin/BAR-specific primers. KO = knockout; ECT = ectopic (B); Southern analysis of ∆modcl1, ∆modcl2 and ∆modcl2/modcl1 mutants. 1kb ladder (1), 70–15 (2), ∆modcl2 KO (3), and Ectopic (5) mutants genomic DNA digested with HindIII, and 70–15 (7), ∆modcl1 KO (8) and Ectopic (11) mutants genomic DNA digested with EcoRI and HindIII (C); ∆modcl2/modcl1 KO1 (2), KO2 (3), Ectopic (4) mutants genomic DNA digested with EcoRI (D). All knock-out mutants were performed in the same way, with the same confirmations. [file 1471-2164-14-326-S10.pptx]

## Slide 1
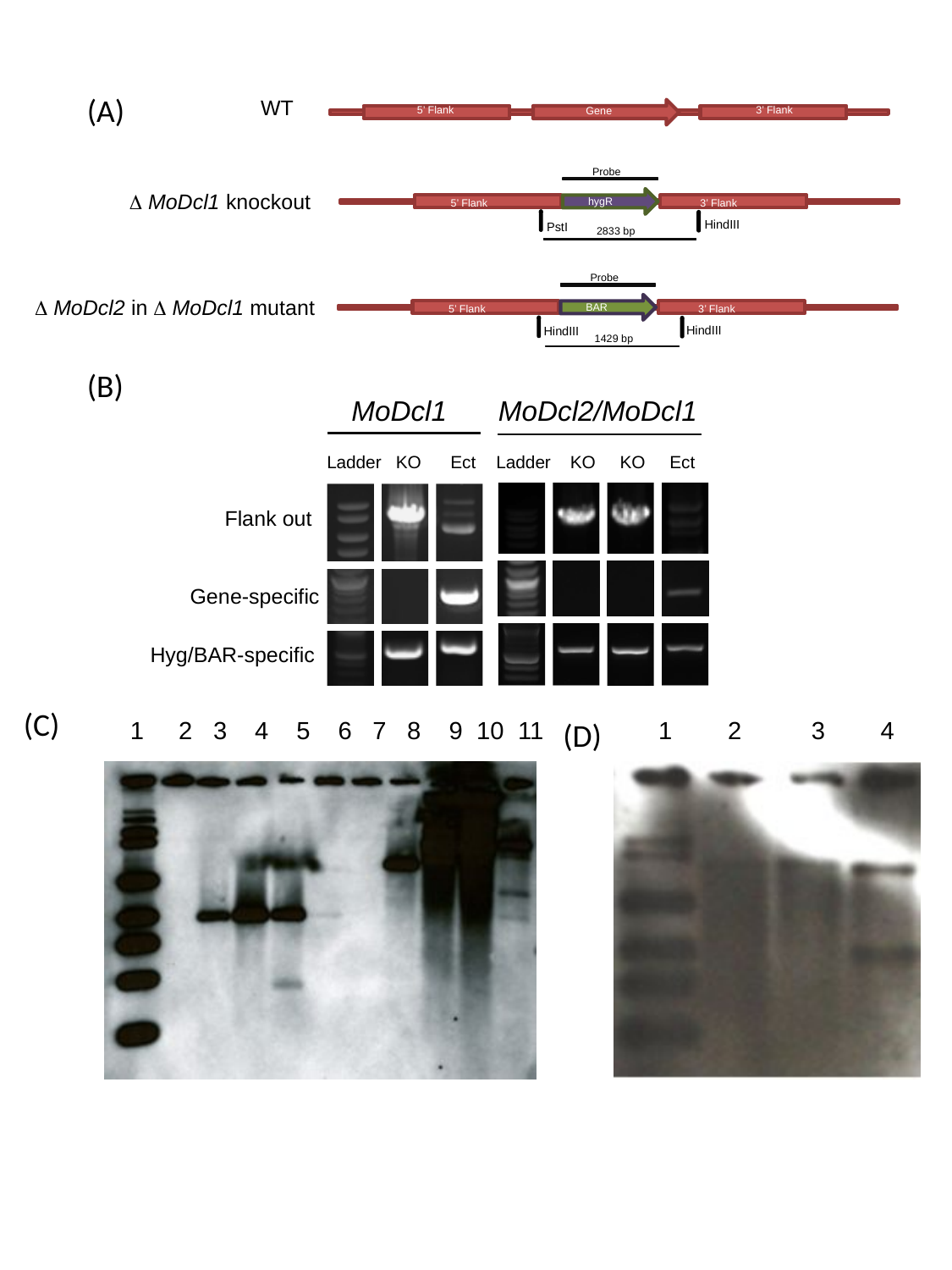

(A)
WT
5’ Flank
3’ Flank
Gene
Probe
hygR
5’ Flank
3’ Flank
HindIII
PstI
2833 bp
Δ MoDcl1 knockout
Probe
BAR
5’ Flank
3’ Flank
HindIII
HindIII
1429 bp
Δ MoDcl2 in Δ MoDcl1 mutant
(B)
MoDcl1
MoDcl2/MoDcl1
Ladder KO Ect
Ladder KO KO Ect
Flank out
Gene-specific
Hyg/BAR-specific
(C)
1 2 3 4 5 6 7 8 9 10 11
(D)
1 2 3 4
